# Nanotechnology-Based Drug Delivery Systems to Control Bacterial-Biofilm-Associated Lung Infections

**DOI:** 10.3390/pharmaceutics15112582

**Published:** 2023-11-03

**Authors:** Yutong Guo, Zeyuan Mao, Fang Ran, Jihong Sun, Jingfeng Zhang, Guihong Chai, Jian Wang

**Affiliations:** 1 School of Pharmaceutical Sciences, Sun Yat-sen University, Guangzhou 510006, China; 2 Department of Radiology, Sir Run Run Shaw Hospital, School of Medicine, Zhejiang University, Hangzhou 310016, China; 3 The Key Laboratory of Diagnosis and Treatment of Digestive System Tumors of Zhejiang Province, Ningbo 315000, China; 4 State Key Laboratory of Respiratory Disease, National Clinical Research Center for Respiratory Disease, Guangzhou Institute of Respiratory Health, The First Affiliated Hospital of Guangzhou Medical University, Guangzhou 510180, China

**Keywords:** chronic lung infections, bacterial biofilm, mucosal barriers, nanoparticle-based drug delivery, biofilm control

## Abstract

Airway mucus dysfunction and impaired immunological defenses are hallmarks of several lung diseases, including asthma, cystic fibrosis, and chronic obstructive pulmonary diseases, and are mostly causative factors in bacterial-biofilm-associated respiratory tract infections. Bacteria residing within the biofilm architecture pose a complex challenge in clinical settings due to their increased tolerance to currently available antibiotics and host immune responses, resulting in chronic infections with high recalcitrance and high rates of morbidity and mortality. To address these unmet clinical needs, potential anti-biofilm therapeutic strategies are being developed to effectively control bacterial biofilm. This review focuses on recent advances in the development and application of nanoparticulate drug delivery systems for the treatment of biofilm-associated respiratory tract infections, especially addressing the respiratory barriers of concern for biofilm accessibility and the various types of nanoparticles used to combat biofilms. Understanding the obstacles facing pulmonary drug delivery to bacterial biofilms and nanoparticle-based approaches to combatting biofilm may encourage researchers to explore promising treatment modalities for bacterial-biofilm-associated chronic lung infections.

## 1. Introduction

Pulmonary infections are the third leading cause of death worldwide; among these, lower respiratory infections account for the top four global causes of death reported by the World Health Organization in 2019. The causative pathogens of pulmonary infections include bacteria, viruses, fungi, or combinations of these [1]. Due to the outbreak of COVID-19, viral pulmonary infections have gained increasing attention in recent years. The main causes of viral lung infections include influenza virus [2], respiratory syncytial virus [3], coronavirus [4], and adenovirus [5], which tend to occur in immunocompromised adults and children [6]. This review focused mainly on bacterial-biofilm-induced lung infections, while viral infections are beyond the scope of this review. Airway mucus usually traps inhaled pathogens and transports them out of the respiratory tract through ciliary beating and coughing [7,8]. However, under some lung disease conditions, such as cystic fibrosis (CF), chronic obstructive pulmonary disease (COPD), and asthma, excessive mucus secretion and/or impaired mucus clearance provide an ideal environment for pathogens to colonize [9]. Most acute bacterial lung infections are amenable to antibiotic treatment, but chronic lung diseases such as CF often result in persistent infections, which are attributed to mucoid bacterial biofilm formation in the sputum [10,11]. Infectious disease experts at the Center for Disease Control and Prevention (CDC) estimated that approximately 65% of human bacterial infections involve biofilms [12]. Bacterial biofilms are communities of sessile microorganisms embedded in a self-produced extracellular polymeric substance (EPS) that physically protects them from the host immune system and antibiotic stress [13,14]. This enhanced resistance to antimicrobial agents is attributed to the restricted penetration of antibiotics, high extracellular enzymatic activity, the decreased metabolism of inner bacterial cells or the presence of persister cells, the expression of specific resistance genes, etc. [15]. Therefore, the biofilm phenotype of bacterial life is responsible for up to a 1000-fold increase in antibiotic resistance [16].

To combat bacterial biofilm in the respiratory tract, researchers have developed various strategies, including a pipeline of new antibiotics, biofilm biomatrix disruption, quorum sensing inhibition, biofilm dispersion promotion, or combinations of these [15]. Among the strategies, nanoparticle-based drug delivery systems have received increasing attention for delivering antibiotics to biofilm sites or enhancing anti-biofilm activity through the nanoparticles themselves [10,15,16,17]. These nanoparticles can be delivered to respiratory biofilm through either intravenous injection or inhalation, and the latter is a more effective and preferred route for patients when treating lung infections. Although inhalation of nanoparticles can cause more antibiotics to be delivered to the respiratory tract, pulmonary anatomy and mucosal barriers strongly restrict the particles’ access to the biofilm site [18,19]. Even though the particles can approach the biofilm site, the barrier rendered by the biofilm biomatrix further limits the access of the antibiotics to the embedded bacteria. Therefore, the accessibility of the nanoparticles to the bacterial biofilm as well as the embedded bacteria is critically important to achieve the goal of biofilm eradication.

Herein, we present a review of the current state of the art concerning the use of nanoparticulate drug delivery systems to inhibit or eradicate respiratory bacterial biofilms. Specifically, the formation of mucosal biofilms in the lungs and the barriers to be considered for nanoparticles to penetrate biofilms and kill bacteria are briefly outlined. Recent advances in nanotechnology-based antimicrobial agents and delivery systems for respiratory biofilm control, in vitro and in vivo lung biofilm models, and the critical factors and prospects for biofilm control using nanoparticulate delivery systems are summarized.

## 2. Respiratory Tract Anatomy and Mucosal Barriers

The respiratory tract is remarkably resistant to environmental attack, despite continuous exposure to pathogens, fine particles, and toxic chemicals in inhaled air. This resistance is attributed to a highly effective defense provided by airway mucus, an extracellular hydrogel lining the lung epithelial surface with a thickness ranging from 10 to 30 μm [20]. Mucus clearance is the primary defense mechanism that protects the airways from inhaled infections and toxic agents. Airway mucus mainly contains water and mucins, which can trap inhaled toxins and transport them out of the respiratory tract by means of ciliary beating and coughing. The airway mucus has two distinct layers, the superficial mucus gel layer, which continuously moves in bulk, and the relatively stable periciliary brush layer [21]. The superficial mucus layer is responsible for the adsorption and capture of inhaled pathogens and particles, and then unidirectionally transports them out of the respiratory tract with the cooperation of ciliated epithelial cells [22]. Normal airway mucus is composed of 97% water and 3% solids, wherein the solids contain mucins, non-mucin proteins, lipids, extracellular DNA, salts, and cellular debris [23]. Mucins constitute less than 30% of the solids, and are predominantly glycosylated, with most terminal sugar chains containing carboxyl or sulfate groups, making them highly anionic. Mucins serve as the primary building blocks of airway mucus networks, formed through reversible associations such as entanglements, hydrogen bonds, and disulfide cross-links facilitated by the terminal cysteine-rich domains. Of these, MUC5AC and MUC5B are the most prevalent in human airway mucus, and their compositions vary based on disease severity [24].

Mucus networks serve as a selectively permeable barrier, permitting the penetration of certain substances, such as nutrients, while restricting the diffusion of foreign toxic agents which might damage the lungs. While mucus gel is continuously propelled by ciliary beating, polymeric mucins are continuously secreted to replenish the mucus gel layer. Consequently, effective airway mucus clearance is essential for lung health. However, respiratory diseases, including asthma, COPD, and CF, are often characterized by obstructed airway mucus and inadequate clearance [9]. The mucus in a healthy airway is a viscoelastic gel, easily moved by ciliary beating. In contrast, pathologic airway mucus typically possesses greater viscosity and elasticity, making it more challenging to clear. The transformation of airway mucus from a healthy to a pathologic state arises from various mechanisms, including alternations in mucus hydration and biochemical components. These changes subsequently affect the rheological properties of the mucus [25,26]. Such modifications can be traced back to the abnormal secretion of water and salts, increased mucin production, and the influx of inflammatory cells into the mucus. Studies have revealed that the solid contents in CF sputum significantly exceed that of healthy airway mucus. Our previous research demonstrated that neutrophil extracellular traps (NETs), released from infiltrating neutrophils in the airway mucus, markedly enhance mucus viscoelasticity and reduce the size of mucus mesh pores [27].

The tenacious mucus lying on the respiratory tract not only shields the lung from harmful environmental toxins but also acts as a transport barrier against drug delivery systems [7]. Airway mucus effectively traps most foreign particles through steric obstruction and/or adhesive interactions, blocking their access to the underlying epithelial surface [28]. These restrained particles are further removed from the mucosal surface, thereby strongly limiting the retention of sustained drug delivery locally. To bypass the airway mucus barrier, the drug-encapsulated particles should be designed to be smaller than the mucus mesh pore size and should exhibit minimal, if any, interaction with the components of the airway mucus [29,30].

## 3. Lung Infections and Bacterial Biofilm Formation

Persistent accumulation of airway mucus in the respiratory tract creates an optimal environment for microbial growth, predominantly leading to lung infections and inflammation. This obstructed mucus further constricts the respiratory passages, establishing a reinforcing cycle that amplifies bacterial proliferation and persistent inflammation. Healthy airway mucus contains antimicrobial peptides, immunomodulators, etc., all of which fortify the host’s defense mechanisms [31]. However, in obstructive pulmonary diseases, changes in pH, ionic strength, and protease activity often impair the efficacy of these antimicrobial peptides. This results in compromised bacterial clearance, heightening the chances of bacterial colonization in the lungs [32]. Compounding this issue, the formation of biofilm within the airway mucus further impairs bacterial eradication, leading to intractable and chronic lung infections. Studies have shown that biofilms account for 60% of chronic infections across 50 documented cases [33]. This aligns with data from the National Institutes of Health, which reported that approximately 80% of chronic microbial infections are biofilm-associated [34]. Microorganisms capable of forming pulmonary biofilm and their related lung infections are summarized in Table 1.

*Pseudomonas aeruginosa* (PA) is one of the most common opportunistic pathogens in hospitals, often linked to nosocomial infections. It stands as a primary concern for immunocompromised patients with COPD, CF, diffused panbronchiolitis, HIV, and cancer, undergoing chemotherapy. *P. aeruginosa* is one of the “superbugs”, which comprise multidrug resistant microbes and a member of the “ESKAPE” panel pathogens [48]. Notably, it is responsible for up to 80% of chronic lung infections in patients with CF. The biofilm of *P. aeruginosa* significantly contributes to its resistance against the host immune response and antibiotic treatment. This resistance is a principal factor behind chronic lung infections in these patients [11,49]. Given the extensive research on the biofilm dynamics of *P. aeruginosa*, it serves as a model to illustrate the stages of biofilm formation, the important role of extracellular polymeric substances (EPS) in the biofilm development, as well as the reasons behind the predominant phenotype of bacterial biofilm in the CF lungs.

### 3.1. The Formation of P. aeruginosa Biofilm

**Figure 1:**
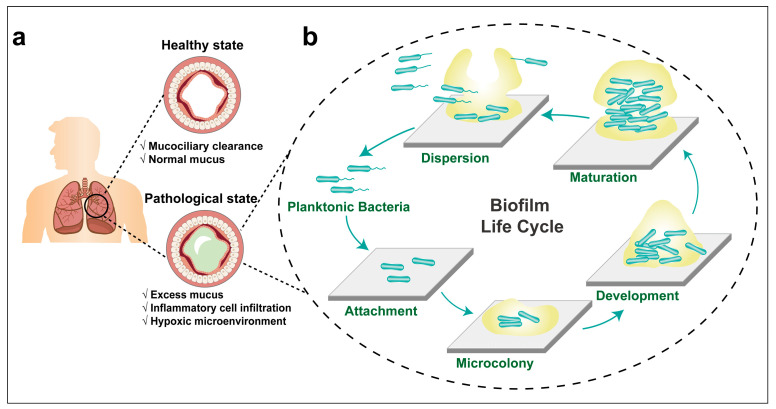
Biofilm formation in pathological lung conditions. (**a**) Schematic illustration of airway mucus alteration under pathological conditions, including asthma, COPD, and CF, which can promote bacteria colonization and growth in the lungs. (**b**) Schematic illustration of the lifecycle of bacterial biofilm. Planktonic bacteria first adhere to the substratum by flagella and hyphae, after which bacteria begin to divide and produce EPS, which provides a substrate for bacterial growth to form a microcolony. Bacterial microcolonies continue to mature and thicken, forming a bacterial biofilm with a certain three-dimensional structure. Upon bacterial biofilm maturation, bacteria disperse from the biofilm and seek new attachment sites to start a new biofilm cycle.

Bacteria exhibit two distinct modes: planktonic and within biofilm. The biofilm mode allows individual planktonic cells to adopt a multicellular growth pattern [50]. Biofilm formation is a developmental process, starting when planktonic bacteria begin to aggregate or transition to a surface-associated mode. The biofilm is completely formed when bacteria escape from the biofilm structure to return to the planktonic mode [51]. It is widely recognized that most bacterial species undergo a cyclic biofilm formation process. Among them, *P. aeruginosa* is the most extensively studied, with its biofilm developmental lifecycle being well documented. The biofilm development cycle can be divided into five phenotypically distinct stages, each regulated in a community paradigm. This process commences when individual planktonic cells make contact with a surface, facilitated by a flagellum. In this sessile growth phase, bacteria cease flagellum gene expression, forming nascent bacteria clusters, and produce polymeric extracellular matrix components. They also secrete virulence factors and develop antibiotic resistance [50]. Bacteria within the biofilm exhibit characteristics markedly different from their planktonic counterparts. Notable traits include enhanced tolerance to various stresses (like dehydration, hypoxia, and starvation), inherent resistance to host immune defenses, and increased resilience to antimicrobial agents. The cyclical nature of bacterial biofilm formation in the respiratory tract is depicted in a stage-by-stage manner in Figure 1.

Attachment: The surface of the lung epithelium is lined with viscoelastic mucus, which serves to lubricate and protect the respiratory tract from inhaled pathogens, fine particles, and toxic materials [52]. The infected free-living planktonic *P. aeruginosa* attach to the mucin, the primary solid component of airway mucus. This adhesion results from interactions between the bacteria’s flagellum and the specific glycoprotein or glycolipid receptors found in the mucus or the respiratory epithelium [53,54]. At this juncture, the adhesion process remains reversible. The attached bacteria can detach and return to the planktonic counterparts again.

Microcolony: Upon attachment, the sessile bacteria begin to form nascent clusters on the substrate. As these clusters grow in both size and density, various extracellular polymeric components, including DNA, lipids, liposaccharides, polysaccharides, and proteins, are excreted under the regulation of quorum sensing (QS) system and hold the bacteria together to form microcolonies [55]. The production of EPS offers a matrix within which the bacteria embed, and the constituents of the EPS further augment the adhesion capability of microcolony [56].

Development: During development, there is an augmentation in the release of extracellular DNA (eDNA) from the bacteria or their debris, which is further enhanced by the highly activated QS system and iron signaling within the bacterial microcolony [57]. Utilizing type Ⅳ pili-mediated DNA binding, a subpopulation of motile bacteria migrates to the core of the microcolony, where non-motile bacteria reside and where eDNA concentration is high. This coexistence of both motile and non-motile bacterial subpopulation within the microcolony contributes to the heterogeneity of *P. aeruginosa* biofilm [58,59].

Maturation: At an appropriate time, microcolonies evolve into true biofilms, which exhibit three-dimensional structures, e.g., a mushroom-shaped colony. The specific three-dimensional structures that emerge vary based on the conditions and environment in which the biofilms develop: mushroom-like structures are common in flow cell systems. Meanwhile, spherical shapes are predominant in more static systems [60]. The carbon source has also been highlighted as affecting biofilm structures. Glucose as a kind of carbon source can form heterogenous mushroom-shaped structures, whereas a uniform and planned structure was found when using citrate as carbon source [61].

Dispersion: The dispersion of bacterial biofilm can be achieved through both passive and native manners of dispersion [50]. Passive dispersion refers to detachment of a biofilm matrix through mechanical or shear stress, such as grazing, abrasion, erosion, sloughing, etc. Continued biofilm growth increases the bacteria crowding and intensifies the chemical gradients, initiating native dispersion. Native dispersion is characterized by bacteria evacuating from the interior portions of cell clusters and forming void spaces. Contrasting passive detachment, bacteria’s escape from three-dimensional structures in native dispersion is an active process. The dispersed bacteria return to a planktonic mode, seeking new sites to restart a biofilm lifecycle; this process is also referred to as “seeding dispersal” [50,62].

### 3.2. The Important Roles of EPS in the Development of Bacterial Biofilm

The bacteria in biofilm live in a self-produced matrix of hydrated extracellular polymeric substances (EPSs) that form their immediate environment [63]. In most biofilms, the polymeric matrix accounts for more than 90% of the dry mass, whereas the microorganisms only account for less than 10%. The EPS predominantly comprises polysaccharides, proteins, lipids, and nucleic acid. Together, they establish a cohesive, three-dimensional polymeric network that interconnects and transiently immobilizes the embedded bacteria. Furthermore, EPS mediates bacteria adhesion to the substratum and ensures the biofilm’s mechanical stability. Given that the EPS composition in a biofilm varies depending on the bacterial species, the dominant components, such as polysaccharides and eDNA, will be discussed in terms of functionality in biofilm formation and progression.

Polysaccharides: Psl and Pel were found to be the two major polysaccharides in biofilm matrices. Psl is a repeating pentasaccharide that comprises D-mannose, D-glucose, and L-rhamnose [64], playing a crucial role in the initial adherence and stabilization of both nonmucoid and mucoid biofilm development [65,66]. Ma et al. visualized the matrix formation in a biofilm lifecycle by staining Psl-specific lectin, demonstrating that Psl is a key scaffolding matrix component, offering potential insights for therapeutics targeting biofilm-related complications [67]. Psl anchors on the host cell via a special helical pattern, enhancing the interaction with adjacent bacteria to stabilize the biofilm structure. During biofilm maturation, a Psl matrix-free cavity emerges within the microcolony, promoting the dispersion of motile bacteria into subsequent stages [67]. Pel stands as another vital polysaccharide in biofilm development, owning to its proficiency in both stabilizing and safeguarding the biofilm structure [68]. A carbohydrate chemical analysis has indicated that Pel is a cationic-charged exopolysaccharide, consisting of partially acetylated N-acetylgalactosamine and N-acetylglucosamine [69]. During biofilm formation, Jennings et al. found that Pel can bind with eDNA via charge interactions by using Pel-specific lectin staining [69].

In *P. aeruginosa* infected patients with CF, the mutation in MucA gene leads to the overproduction of alginate, which is significant for the transition of *P. aeruginosa* from nonmucoid to mucoid phenotype [63]. Furthermore, the elevated production of alginate correlates strongly with chronic infections in patients with CF, given that *P. aeruginosa* shows increased expression of genes encoding both alginate and Psl polysaccharides in CF lungs [70]. Alginate plays a role in microcolony formation and in stabilizing the mature biofilm structure [63]. Moreover, alginate can exacerbate lung infections by inhibiting the phagocytosis of macrophages and neutrophils, suppressing lymphocyte function, and triggering inflammatory response. All of these factors contribute to promoting the formation of *P. aeruginosa* biofilm [71]. Nonetheless, alginate is not a necessary component for biofilm formation. Wozniak et al. observed that commonly used experimental strains of *P. aeruginosa*, PAO 1 and PA 14, form a nonmucoid biofilm without producing alginate. Since alginate is a specific component of the *P. aeruginosa* mucoid biofilm matrix, researchers have suggested that an alginate-specific antibody might be effective for use in diagnosing *P. aeruginosa*-biofilm-associated lung infections [72]. Consequently, alginate lyase has been employed to disrupt the *P. aeruginosa* biofilm matrix and combat related infections [73,74,75]. Collectively, Psl, Pel, and alginate each play pivotal roles in *P. aeruginosa* biofilm adhesion, development, and stability. Beyond these roles, these polysaccharides shield the resident bacteria from antimicrobial treatments and host immune defenses, rendering bacterial-biofilm-associated chronic infections particularly challenging to address [76].

Extracellular DNA (eDNA): eDNA is another important component in the *P. aeruginosa* biofilm matrix throughout biofilm development. eDNA is derived from cell lysis and the outer membrane vesicles (OMVs) of bacteria [77]. eDNA release is regulated by QS systems, complemented by other regulators such as pyocyanin and iron signals [77,78]. Negatively charged eDNA can interact with divalent cationic metal ions, such as Mg^2+^ and Ca^2+^, to stabilize biofilm structures, whereas these chelates increase resistance to antimicrobial agents [79]. Furthermore, eDNA acts as matrix glue to crosslink with Pel and Psl polysaccharides, forming the biofilm structure skeleton [69,80]. It is noteworthy that biofilm eDNA facilitates swift electron transfer amidst redox-active intercalators, thereby sustaining the metabolic activity of specific bacterial populations within multicellular biofilm [81]. Functionally, biofilm eDNA acts as a shield, protecting *P. aeruginosa* against aminoglycosides treatment [82]. In CF airway mucus, there is abundant eDNA generated from necrosis neutrophils [77], and this eDNA can further activate human neutrophil to trigger host inflammatory response [83]. Additionally, *P. aeruginosa* has been found to utilize these eDNA to build biofilm structure, enhancing bacterial survival in humans [80].

Besides polysaccharides and eDNA, numerous other essential components are involved in biofilm development. In CF airways, rhamnolipid, a kind of virulence factor, can inactivate tracheal cilia, complicating treatment for affected patients [84]. As an excellent biosurfactant, rhamnolipid can reduce surface tension and promote bacterial movement across the semisolid surface through flagellum-based propulsion [84]. Moreover, rhamnolipid has been reported to affect biofilm structures and induce bacteria dispersion in a concentration-dependent manner [85,86].

### 3.3. P. aeruginosa Biofilm-Associated Lung Infections in Cystic Fibrosis Patients

Cystic fibrosis (CF) is a hereditary condition stemming from mutations in the cystic fibrosis transmembrane conductance regulator (CFTR) gene. This mutation predominantly impacts the respiratory tract, gastrointestinal system, and reproductive organs [87]. The malfunction of chloride channel in the lung caused by CFTR gene mutation results in an abnormal hydrated periciliary liquid layer, culminating in the production of viscous and dense mucus [88]. Accumulation of this tenacious mucus in the airway attenuates mucociliary clearance, which in turn facilitates the colonization of bacteria in the mucus layer [88].

*P. aeruginosa* predominantly colonizes stagnant mucus in CF airways, and this bacterium readily forms mucoid biofilms. Upon *P. aeruginosa* invasion, there is a marked recruitment of polymorphonuclear leukocytes (PMNs) and alveolar macrophages to the infection site. PMNs produce a metabolic burst by releasing large amounts of reactive oxygen species (ROS) during phagocytosis of bacteria. The ROS not only neutralize bacteria, but also inflicts damage upon host tissues [89]. Intriguingly, the ROS generated by PMNs is believed to trigger mutations in the MucA gene, leading to excessive alginate production [63]. The alginate can scavenge oxygen radicals and protect against bacterial phagocytosis and free radical damage [89]. As mentioned previously, the overproduction of alginate plays a pivotal role in the transition from nonmucoid to mucoid phenotypic biofilm. Collectively, the mucoid phenotypic biofilm shields the embedded bacteria, making their elimination by the host immune system challenging. This results in persistent and hard-to-treat infections, complicating clinical interventions.

Chronic lung infections and inflammation in CF lungs is often accompanied by neutrophils infiltration, and the necrosis of these neutrophils produces large amounts of eDNA [77]. *P. aeruginosa* has been found to utilize DNA to build biofilm structures. In addition, actin produced by the necrotic neutrophils has been reported to serve as an attachment site for biofilm development [90]. Notably, our previous research emphasized that neutrophil extracellular traps (NETs), released upon neutrophil stimulation, contribute to airway mucus obstruction through increasing mucus viscoelasticity, on account of DNA release and oxidative stress [27]. These infiltrated neutrophils in CF airways play important roles in the formation of biofilm and enhance bacterial survival, making them a potential focal point for biofilm disruption and elimination.

The viscous mucus gel layer blanketing airway epithelium creates an anaerobic or microaerophilic environment for *P. aeruginosa* proliferation [89]. As a facultative anaerobe bacterium, *P. aeruginosa* prefer anaerobic respiration of nitrate or nitrite in hypoxic conditions [91]. Functional annotation of *P. aeruginosa* CF isolates revealed that these clinical strains have a unique expression profile of replication, membrane biogenesis, and virulence proteins during hypoxia, indicating the diversity of the mechanisms held by these bacteria to adapt to low-oxygen environments and initiate robust molecular responses to persist under hypoxic stress [92]. In addition, *P. aeruginosa* has been found to alter the structure and physicochemical properties of rhamnolipids, essential EPS components, in the absence of oxygen [93]. *P. aeruginosa* has also been found to regulate the expression of the Anvm-protein to impact its pathogenicity and host defense under hypoxic conditions [94]. Consequently, *P. aeruginosa* possesses a remarkable ability to acclimate to the oxygen-poor environment of CF airways, aptly positioning it to flourish within the hypoxic mucus plugs characteristic of CF lungs.

Compromised mucociliary clearance among patients with CF is another reason for the ready colonization of *P. aeruginosa* in the airways; the components in the sputum in addition to oxygen deficiency facilitate the formation of mucoid biofilm. Once biofilm has formed, bacteria embedded in the matrix can be protected and become less susceptible to host immune systems and antibiotic stress, which severely impedes bacterial clearance and lung infection treatment. The subsequent sections delve into the mechanisms underlying the resistance of bacterial biofilms.

## 4. Mechanisms of Antibiotic Resistance of Biofilm Bacteria

**Figure 2:**
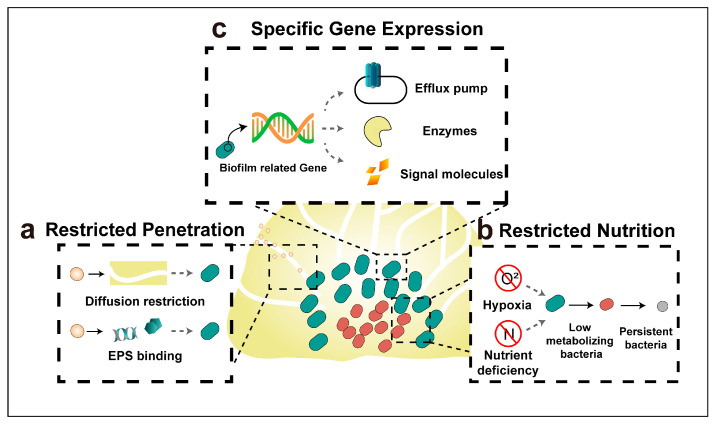
Mechanisms underlying antibiotic tolerance in bacterial biofilm. (**a**) The biofilm acts as a barrier and prevents the penetration of antibiotics through diffusion restriction and adhesion. (**b**) Within biofilms, the presence of persistent bacteria is heightened due to limited nutrient availability. (**c**) Bacteria within biofilms express specific genes that enhance their resistance to antibiotics.

Bacteria within biofilms demonstrate antibiotic tolerance levels up to 1000-fold higher than their planktonic counterparts [60,95]. Here, the tolerance that is referred to is not exactly the same as the concept of bacterial resistance; rather, it denotes a reversible and adaptive phenotype of bacteria [96]. Upon dispersion, the bacteria derived from biofilm regain their susceptibility to antibiotics, in contrast to bacterial resistance, which operates to prevent interactions between antibiotics and their target sites [97]. The tolerance of bacterial biofilm to antibiotics is caused by joint efforts of restricted drug penetration, physiological shielding, and the expression or modification of specific genes (Figure 2); the tolerance mechanisms will be discussed in detail hereafter.

### 4.1. Restricted Penetration by The Biofilm Matrix

The three-dimensional architecture of biofilms can impede the diffusion of antibiotics, leading to a reduced concentration of antibiotics within the biofilm matrix [97]. Moreover, various components of the biofilm matrix can bind with antibiotics, further inhibiting their penetration into biofilm. Negatively charged eDNA, an indispensable component of bacterial biofilm matrix, was found to bind with positively charged aminoglycoside antibiotics through electrostatic interaction, thus preventing their biofilm penetration. Biofilm formed by DNA-release deficient *P. aeruginosa* were shown to be more susceptible to aminoglycoside antibiotics compared to those formed by wild-type *P. aeruginosa*, and the biofilm formed by this mutant strain restored its tolerance to aminoglycoside upon the addition of exogenous DNA.

### 4.2. Restricted Nutrition and Emerging of Persister Bacteria

There exists a steep gradient of oxygen and nutrients from the surface to the inner of the biofilm; thus, the inner bacteria have lower metabolic activity than the superficial ones. Since most of the antibiotics target the growth processes of bacteria (e.g., bacterial cell wall synthesis, protein synthesis, and nucleic acid replication and transcription), these inner, slower-metabolizing bacteria were found to have heightened antibiotics tolerance [11]. Although combining aminoglycosides, polymyxins, and fluoroquinolones can enhance antibacterial efficacy, a residual population of bacteria, termed “persister bacteria”, still survives [60]. Challenged by both nutrient depletion and antibiotic stress, persister bacteria are recognized to be closely associated with chronic and recurrent infections [98]. A prime example is *Mycobacterium tuberculosis*, which can endure multi-antibiotic treatments and remain in a prolonged latent state [99].

### 4.3. Specific Gene Expression

In contrast to their planktonic counterparts, bacteria within the biofilm can express specific genes that enhance bacteria tolerance [11]. For instance, *Burkholderia pseudomallei*, responsible for melioidosis, has been reported to tolerance to ceftazidime (CAZ) in its biofilm state, while remaining sensitive in its planktonic state [100]. RNA-sequencing studies of this bacterium across planktonic, biofilm, and planktonic shedding states revealed that approximately 10% of gene expression changed in both biofilm and planktonic shedding compared to planktonic bacteria. The enhanced tolerance of the biofilm to CAZ is believed to be associated with nitrite stress response, iron–sulfur homeostasis, and nitrate respiration. Biofilm-based bacterial tolerance can be either general or specific to certain antibiotics, e.g., c-di-GMP has been reported to be related to the nonspecific tolerance of *P. aeruginosa* biofilm to a variety of antibiotics. Poudyal et al. discovered that the PA3177 gene encodes a diguanylate cyclase influencing the c-di-GMP level in the biofilm, and deactivating this gene improves biofilm susceptibility to tobramycin treatment [101]. Besides, BrlR, c-di-GMP responsive transcriptional regulator, can boost the expression of both ABC transport system genes and multi-drug efflux pump genes, intensifying the biofilm’s tolerance to antibiotics [102]. Elevated β-lactamase transcription level in *P. aeruginosa* biofilm is a specific example of magnifying tolerance to β-lactam antibiotics [60].

In summary, given the protective nature of biofilms, conventional antibiotics often fall short in treating biofilm-associated infections. As a result, there is a pressing need to develop innovative therapeutic strategies to effectively tackle bacterial biofilm. Nanotechnology-based antimicrobial drug delivery systems have increasingly come into the spotlight owning to their unique physicochemical properties. Antimicrobial nanomedicine possesses small particle size, facilitating deeper penetration through both mucus and biofilm matrices. A variety of nanoparticles can not only encapsulate antibiotics efficiently but also inherently exhibit antimicrobial activity. In the following sections, we will delve into the latest advances in nanotechnology-based antimicrobial drug delivery systems and their pros and cons in treating biofilm-associated lung infections.

## 5. Administration Routes of Nanomedicines for the Treatment of Lung Infections

### 5.1. Systemic Administration

The judicious design of nanoparticles can enable their effective accumulation at the infected site upon systemic administration, thereby minimizing systemic side effects. Various strategies, such as microenvironmental response [103], ultrasound response [104], inflammation targeting [105], and photodynamic and photothermal stimulation [106], can be employed to enhance nanoparticle penetration and retention at the infection site.

### 5.2. Inhalation Administration

Inhalation presents an ideal route for treating pulmonary infections, allowing the drug to be delivered directly to the infection site and significantly reducing the required drug dose. Nevertheless, the presence of the airway mucus barrier, macrophage phagocytosis, and pulmonary surfactant can impede the efficiency of pulmonary delivery [107,108]. By judiciously designing nanoparticles, one can enhance the efficiency of nanomedicine delivery to the lungs.

#### 5.2.1. Strategies to Overcome Airway Mucus

The airway mucus barrier arises primarily from the adhesive properties of mucus components and the diffusion barrier created by the mucin network. The average pore size of the human airway mucin network is roughly 200 nm [109]. Under pathological conditions, when the mucus thickens, the pore size of the mucin network decreases [110]. Nanoparticles exceeding the mucin network’s pore size face difficulty traversing this mucus barrier. Given the abundance of negatively charged components in mucus, positively charged nanoparticles could be immobilized within the mucin network by electrostatic interaction. Zhao et al. [111] investigated the in vivo behavior of positively, negatively, and neutrally surface-charged liposomes post inhalation. The findings revealed that both neutrally and negatively charged liposomes exhibited superior mucus permeability, diminished macrophage uptake, prolonged intrapulmonary retention, and reduced exposure to other organs. Inspired by viruses, researchers found that amphiphilic ionic nanoparticles also reduced the binding of mucus components to the nanoparticle surface [112]. Beyond electrostatic interactions, hydrophobic interactions can also hinder nanoparticle diffusion through airway mucus. Consequently, nanoparticles possessing a hydrophilic surface typically exhibit superior mucus penetration compared to their hydrophobic counterparts [113]. Additional strategies enhancing mucus penetration will be elaborated upon in subsequent sections.

#### 5.2.2. Strategies to Avoid Macrophage Phagocytosis

Alveolar macrophages play a pivotal role in clearing nanoparticles that deposit in the airway, with this clearance mechanism being significantly influenced by factors like particle size, shape, and surface properties, etc. Chono et al. [114] investigated the impact of pulmonary administration of liposomes of varying sizes (100, 200, 400, 1000, and 2000 nm) on their endocytosis through alveolar macrophages. The results indicated that the uptake of liposomes by alveolar macrophages augmented as the particle size increased from 100 to 1000 nm, stabilizing when the size exceeded 1000 nm. Moreover, the existence of negatively charged sialic acid on macrophage surfaces was found to facilitate the electrostatic binding of positively charged nanoparticles to macrophages, subsequently enhancing phagocytosis [115].

#### 5.2.3. Interaction with Pulmonary Surfactants

Pulmonary surfactant, a complex of phospholipids and proteins, is synthesized and secreted by type II alveolar epithelial cells. When inhaled nanoparticles reach the deeper regions of the lungs, they encounter pulmonary surfactants that can adsorb onto their surface, forming a biomolecular corona. This corona significantly influences the stability and cellular uptake patterns of the nanoparticles, acting as a primary determinant of the biological behavior and fate of nanomedicines within the lungs [116]. The particle size, surface charge, and hydrophilic or hydrophobic properties of inhaled nanoparticles are key parameters that impact the interaction between these nanoparticles and pulmonary surfactants [117].

## 6. Nanotechnology-Based Diverse Antimicrobials for Bacterial Biofilm Control

**Figure 3:**
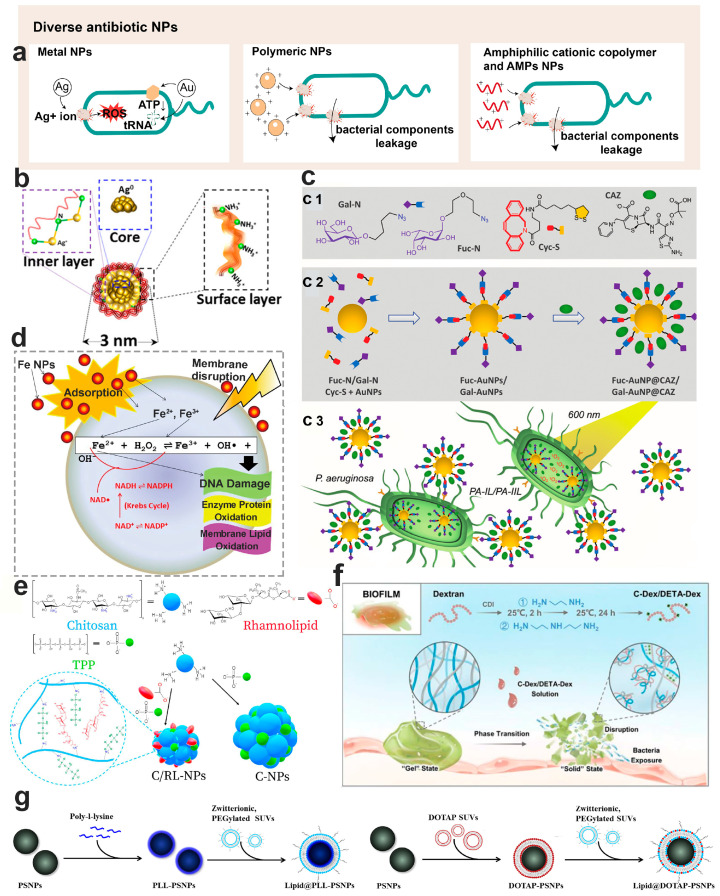
Diverse antibiotic nanoparticles employed to combat bacterial biofilm. (**a**) Various antibacterial mechanisms of different nanomaterials; (**b**) diagram illustrating antibacterial Ag NPs (Ref. [135]); (**c**) glycoconjugate-based Au NPs developed for targeted treatment against *P. aeruginosa* biofilm (Ref. [141]), where c1 depicts the essential building blocks for creating of Fuc-AuNP@CAZ and Gal-Au@CAZ, c2 illustrates the one-pot self-assembly procedure and simultaneous integration of Ceftazidime, resulting in Fuc-AuNP@CAZ/Gal-Au@CAZ, and c3 represents the targeted lectin approach of Fuc-AuNP@CAZ/Gal-Au@CAZ, which selectively enters *P. aeruginosa* to concurrently release the drug and generate heat/ROS when exposed to photoirradiation; (**d**) mechanisms underlying bacterial damage post exposure to iron NPs (Ref. [151]); (**e**) a schematic representation of the synthesis of both chitosan NPs and chitosan/rhamnolipid NPs (Ref. [160]); (**f**) a diagram illustrating the disruption of bacterial biofilm by cationic dextran through the phase transition (Ref. [165]); (**g**) schematic representation of the preparation of Lipid@PLL-PS NPs and Lipid@DOTAP-PS NPs using the electrostatically driven layer-by-layer method (Ref. [156]).

Nanotechnology-based antimicrobials represent promising arsenals that have been used to deliver antibacterial agents, effectively targeting planktonic bacteria, antibiotic-resistant strains, and biofilm-forming bacteria. When encapsulated within nanoparticles, antibiotics not only retain their antibacterial activity but also leverage the unique properties of nanoparticles, further amplifying anti-biofilm actions. Such antibiotic-loaded nanoparticles can be further optimized to enhance their anti-biofilm efficacy. Typically, the size of these nanoparticles is smaller than the size of the airway mucus network, enabling them to effortlessly penetrate both the airway mucus and biofilm structures. The new insight into the application of nanoparticles against bacterial biofilm will be reviewed as follows in terms of their types (Figure 3a), design strategies, and biofilm-combating mechanisms.

### 6.1. Physicochemical Properties of Anti-Biofilm Nanoparticles

The physicochemical properties of nanomedicines can significantly influence their efficacy in biofilm control. Biofilms often possess a complex three-dimensional matrix that can hinder nanoparticles from reaching embedded bacteria [97]. To ensure effective penetration, the size of nanoparticles should be smaller than the dimensions of the water-filled channels within biofilms. Multiple studies have suggested that the ability of nanoparticles to penetrate biofilms diminishes as their diameters increase [118,119]. Nonetheless, for intravenous administration, nanoparticles with a diameter below 5 nm might be readily cleared from the body. Importantly, factors such as nutrient conditions and fluid shear presence can lead to variations in the structure of the biofilm matrix, even within the same bacterial species. Additionally, biofilm matrix components differ among various bacterial types. Generally, for effective biofilm control, a nanoparticle diameter below 200 nm is recommended.

Regarding the systemic administration of nanomedicines, the surface charge of the nanoparticles plays a pivotal role in ensuring effective delivery to infectious biofilms. Positive surface charges can enhance bacteria-killing effects through contact-mediated lethal membrane damage and subsequent cell death. Nevertheless, nanoparticles with positively charged surfaces can become targets for opsonization and immune cells recognition, complicating their journey to infected sites via blood circulation [120]. A recent trend suggests using charge-reversible nanoparticles, which balance the stealthy transport properties in blood while retaining their bacteria-killing capabilities.

Beyond size and surface properties, the shape of the nanoparticles also impacts their anti-biofilm effectiveness. Certain shapes enable nanoparticles to adhere more tightly to bacteria, boosting the efficacy of positively charged nanoparticles. Those with sharp edges can compromise bacterial membrane integrity, leading to cellular content leakage and eventual cell death [121,122]. Furthermore, the shape of nanoparticles affects their in vivo behavior, including hemorheological dynamics, cellular uptake, and blood circulation half-life [120].

### 6.2. Inorganic Antimicrobial Nanoparticles

Inorganic materials, with their distinct physiochemical properties, have been harnessed for centuries. Notably, specific types of inorganic nanoparticles demonstrate potent antimicrobial actions, primarily through ROS generation, DNA damage, and cell membrane destruction. Enhancing these nanoparticles with surface functionalization, via polymers, small ligand molecules, and charged moieties, allows for drug encapsulation and controlled release.

Inorganic nanoparticles are typically prepared by top–down and bottom–up approaches. The top–down approach involves converting larger particles into their nano-sized counterparts, whereas the bottom–up approach constructs nanoparticles from atoms or molecules [17]. Concerning synthesis, these approaches can be categorized into physical, chemical, and green synthesis techniques. Physical methods, including laser ablation, ball milling, ion sputtering, and ultraviolet radiation [123,124], primarily use the top–down approach and often necessitate intricate apparatus and specific conditions. On the other hand, chemical methods such as hydrothermal methods, electrochemical synthesis, sol-gel synthesis, the co-precipitation method, and microemulsion synthesis [125] are quicker and more adaptable. However, the potential toxicity and challenging removal of reagents involved limit their applicability.

Green synthesis, which utilizes plants, bacteria, fungi, or their derivatives as raw materials, is gaining traction due to its sustainable nature and the absence of toxic byproducts [17]. Various plant components, such as polysaccharides, amino acids, enzymes, phenols, and saponins, act as reducing and stabilizing agents, giving rise to inorganic nanoparticles of diverse shapes and sizes. For example, various plant parts, including seeds, fruits, flowers, and roots, can be used for Ag NPs synthesis [126]. Beyond plant sources, microorganisms like algae, fungi, bacteria, and yeast are also employed extensively for nanoparticle synthesis. This microbial synthesis can either be intracellular, where metal ions attach to cell walls via electrostatic forces and undergo enzymatic reduction, or extracellular, which involves microbial metabolites (e.g., lipids, proteins, pigments) serving as capping and reducing agents, simplifying subsequent purification processes [127,128]. A study by Kashyap et al. [129] demonstrated that treating Scenedesmus sp. MT636554 cells with AgNO_3_ (0.5 mM and 1 mM) led to the biosynthesis of intracellular Ag/AgCl nanoparticles ranging from 10–50 nm, exhibiting strong antibacterial activity against four bacterial strains.

#### 6.2.1. Ag Antimicrobial Nanoparticles

Ag nanomaterials have been recognized as a promising antimicrobial agent for decades. Their antimicrobial mechanisms include the following: (1) disruption of the bacterial cell wall and membrane structure; (2) damage to subcellular structure [130,131]. Ag NPs have been shown to interact with peptidoglycan on the bacterial cell wall, either directly via individual NPs or through the Ag^+^ ions released from the NPs, leading to bacterial wall disruption [132]. In addition, Ag NPs or the released Ag^+^ ions were found to interact with the biomolecules on the bacterial membrane, notably lipopolysaccharides, causing membrane damage and subsequently cell lysis [133]. Once Ag NPs are endocytosed into the bacterial cytoplasm, the subsequently released Ag^+^ ions emerge as the key factor in driving the antimicrobial activity [134]. Reactions causing oxidation on the Ag NPs’ surface initiate the release of Ag^+^ ions, subsequently leading to the generation of reactive oxygen species (ROS). Elevated levels of Ag^+^ ions and ROS have been implicated in damaging DNA, proteins, and other subcellular structures, which disrupts bacterial metabolism and leads to bacterial lysis [130].

The physiochemical properties of Ag NPs, including their size, oxidation states, and surface coating, influence the release of Ag^+^ ions. Smaller-sized Ag NPs are thought to release Ag^+^ ion quicker than their larger counterparts; this characteristic is attributed to their larger surface area [134]. Therefore, Ag nanoclusters (Ag NCs), defined as ultrasmall NPs with a core of “countable” Ag atoms shielded by surrounding organic ligands, have been developed as new generation of Ag antimicrobial agents [130]. Typically, Ag NCs have a particle size ranging between 1 and 2 nm, a trait that significantly bolsters their antibacterial and antibiofilm properties. Haidari et al. designed an ultrasmall, uniform, and polycationic Ag NC variant for biofilm eradication (Figure 3b). Notably, the high percentage (>50%) of Ag^+^ nano-reservoirs on these clusters grants an enhanced ability to Ag NCs to penetrate the bacterial cell membrane and significantly delay the onset of bacterial resistance compared to similarly sized negatively charged counterparts or conventional antibiotics [135]. Although Ag NPs and Ag NCs showed great potential in combating bacterial biofilm, their intrinsic toxicity remains a major obstacle to their clinical application. Amyloid, a recently identified target for bacterial biofilm, has mechanisms of inhibition that remain elusive [136,137,138]. Huma et al. explored the potential of Ag NPs and Ag NCs at sub-bactericidal concentrations to hinder functional amyloid formation, thereby curtailing biofilm genesis. Their findings suggest that both agents hold promise for development as effective anti-biofilm materials.

#### 6.2.2. Au Antimicrobial Nanoparticles

The antimicrobial mechanisms of Au nanomaterials include the following: (1) binding to the bacterial membrane to alter its membrane potential; (2) reducing the ATP level by inhibiting ATPase activity, leading to declined metabolism; (3) preventing the binding of tRNA with ribosome, potentially inhibiting biological processes [139,140]. Unlike other metal nanoparticles, Au NPs do not induce ROS generation, rendering them a much safer metal antimicrobial agent [140]. Additionally, functionalization can further amplify their antimicrobial and antibiofilm efficacy. Inspired by the selective carbohydrate-based recognition of the key virulence factors of *P. aeruginosa*, namely LecB (fucose-specific lectin) and LecA (galactose-specific lectin), Zhang et al. designed fucose-based (Fuc-AuNPs) and galactose-based (Gal-AuNPs) glycoconjugate Au NPs (Figure 3c) [141]. Both Fuc-AuNPs and Gal-AuNPs exhibited notable bacterial targeting efficiency. When loaded with ceftazidime, these nanoparticulate carriers exhibited both photothermal and photodynamic therapeutic effects, achieving impressive biofilm eradication.

Au NPs have been widely used in photothermal therapy (PTT) for treating cancer and infection, attributed to their exceptional light-thermal conversion efficiency under external light source illumination. The PTT strategy can curb the proliferation of drug-resistant bacteria and disrupt the structure of biofilm, positioning it as a potent strategy for biofilm control [142]. Cui et al. designed a near-infrared (NIR) light-driven nano-swimmer (HSMV) with asymmetrically embedded Au NPs. This innovation efficiently self-propels and penetrates *S. aureus* biofilm within 5 min under NIR light irradiation [143]. The thermal-triggered release of co-loaded vancomycin from this HSMV achieved an efficient combination of photothermal therapy and chemotherapy in one system. While some reports stated that Au nanomaterials can attain temperatures close to 70 °C under illumination [144,145,146], posing a challenge for photothermal therapy, more recent research suggests that the localized temperature for such therapy can be maintained at around 45 °C [143,147]. This optimization ensures effective bacterial treatment while minimizing harm to surrounding tissues.

#### 6.2.3. Metal Oxide Antimicrobial Nanoparticles

Metal-oxide-based antimicrobial nanoparticles typically comprise materials like ZnO [148], CuO [149], Fe_3_O_4_ [150], etc. Their antimicrobial mechanisms can be summarized as follows: (1) disruption of bacterial cell wall and membrane structure, which leads to the leakage of intracellular substances and subsequent bacterial death; (2) generation of ROS, inducing oxidative stress; (3) releasing of toxic metal ion [139]. Armijo et al. investigated the inhibitory concentration and susceptibility of iron–oxide (Fe_3_O_4_) NPs (Figure 3d), both in combination with and without tobramycin, against *P. aeruginosa*. Their findings highlighted that the capping agent of the Fe_3_O_4_ NPs critically influences its bactericidal capabilities. For instance, particles capped with PEG showed no susceptibility, while those capped with alginate demonstrated enhanced dispersibility in alginate-rich biofilms, leading to improved diffusion through bacterial biofilm barriers [151]. The influence of the magnetic field on the performance of Fe_3_O_4_ NPs was further investigated in an in vitro model that utilized an artificial biofilm (alginate layer) and mucus (ager layer). Under an external magnetic field, the bactericidal effect of the Fe_3_O_4_ NPs, both with and without alginate capping, was enhanced. Moreover, while the susceptibility of tobramycin against the bacteria diminished over time, the susceptibility of Fe_3_O_4_ NPs remained consistent, suggesting their potential as promising antimicrobial agents.

In summary, metal nanoparticles offer great potential as anti-biofilm agents through various mechanisms, including metal ion release, bacterial structure disruption, and ROS generation. However, it is crucial to also consider the limitations of these antimicrobial nanoparticles. For instance, Ag NPs catalyze electron transfers that consume oxygen molecules, triggering a cascade of radical reactions and ROS generation. This process can subsequently lead to oxidative stress and cellular malfunctions in organs such as the kidneys, liver, lungs, brain, and spleen [152]. Specifically, Ag NPs of 20 nm in size have been shown to induce lung eosinophil and neutrophil inflammation, paired with bronchial hyper-responsiveness [152]. Furthermore, emerging adaptions and cross-resistances in bacteria and their biofilms to inorganic antimicrobial NPs are concerning. Bacteria in the biofilm have been found to evolve a reduced sensitivity to Ag NPs due to the evolution of persist bacteria within these structures. Cross-resistance between Ag and antibiotics, as seen in gentamicin-resistant *P. aeruginosa* biofilm [153], has also been observed. Therefore, before transitioning these nanomedicines to clinical applications, it is imperative to delve deeper into their toxicity and the bacterial resistance mechanisms they may induce.

### 6.3. Polymeric Antimicrobial Nanomaterials

Polymeric antimicrobial nanoparticles, including lipid and polymer nanoparticles, are viewed as powerful platforms for overcoming biofilm resistance. These particles can enhance the delivery of antibiotics to biofilm-residing bacteria, thus amplifying the efficacy of biofilm eradication. In this section, we provide a comprehensive overview of various types of lipid and polymer nanoparticles that have been developed for combating bacterial biofilm caused infections. Moreover, we also delve into functionalization and targeting strategies crafted to enhance their antimicrobial prowess.

#### 6.3.1. Lipid-Based Antimicrobial Nanoparticles

Lipid-based nanoparticles, such as liposomes, solid lipid nanoparticles, etc., have been employed in drug delivery for decades due to their excellent biocompatibility and safety. Certain cationic lipid materials, such as 1,2-dioleoyl-trimethylammonium-propane (DOTAP), can be integrated into lipid–polymer hybrid nanoparticles (LPNs). This allows the LPNs to attach to the surfaces of both Gram-positive and Gram-negative bacteria, regardless of their planktonic or biofilm lifestyles, thus demonstrating enhanced bactericidal activities. Traditional techniques for producing lipid-based nanoparticles include methods such as thin-film hydration, the freeze–thaw method, high-pressure homogenization, membrane extrusion, reverse-phase evaporation, and solvent injection method, among others. In terms of scaling up production, the microfluidic technique has been employed in nanomedicine manufacturing. This process creates nanoparticles by skillfully handling minuscule fluid amounts within microchannels, significantly bolstering the control and consistency of the nanoparticles [154]. Baek et al. designed a non-toxic LPNs delivery system, consisting of a polymer core (PLGA) and cationic lipid shell (DOTAP). These LPNs, with their uniform particle size, encapsulated over 95% of the antibiotic [155]. Impressively, these LPNs decreased biofilm activity by more than 95% at concentrations 8–32 times lower than free antibiotics, attributed to the targeted and prolonged release of antibiotics at the biofilm site. This suggests that LPNs could be an effective nanocarrier to augment biofilm treatment. By fusing DOTAP-modified polymeric NPs with zwitterionic unilamellar vesicles, Wan et al. designed lipid-bilayer-enveloped lipid–polymer hybrid nanoparticles aimed at treating *P. aeruginosa* biofilm caused lung infections (Figure 3g) [156]. These lipid–polymer hybrid NPs, composed of a polystyrene core and modified with the positively charged DOTAP and poly-L-lysine (PLL) as the shell, were then enveloped in zwitterionic unilamellar vesicles made of 1,2-dioleoyl-sn-glycero-3-phosphocholine (DOPC) and 1,2-distearoyl-sn-glycero-3-phosphoethanolamine-N-[methoxy(polyethylene glycol)-2000] (DSPE-PEG2000). The inclusion of DSPE-PEG2000 in the outer membrane enhanced the mucus penetration capabilities of these particles, ensuring efficient access and penetration into the biofilm. Additionally, DOPC played a pivotal role in aiding these particles to merge with bacterial membranes, positioning this nanocarrier as a potent solution to tackle the persistent challenges of mucus and biofilm barriers.

#### 6.3.2. Chitosan Antimicrobial Nanoparticles

Chitosan is a biocompatible and biodegradable polysaccharide composed of glucosamine and N-acetyl-glucosamine residues. The amine groups of glucosamine give chitosan its cationic properties, enabling it to interact with the negatively charged components on bacterial membrane, such as the teichoic acid in Gram-positive bacteria and the lipopolysaccharide of Gram-negative bacteria. This interaction can lead to cell membrane damage and leakage of intracellular components. Moreover, chitosan has been found to bind with bacterial DNA, consequently inhibiting mRNA transcription and protein synthesis [157]. The prevalent techniques for preparing chitosan nanoparticles include methods like ionic gelation, microemulsion, polyelectrolyte complexation, emulsification solvent diffusion, and the reverse micellar method [158]. Ma et al. encapsulated curcumin into positively charged chitosan nanoparticles, and these curcumin-loaded nanoparticles exhibited strong antibacterial activity against biofilm formed by planktonic bacteria or fungi, irrespective of whether they were single or polymicrobial organisms [159]. Rhamnolipid, a natural glycolipid known for its antimicrobial, anti-adhesive, and biofilm-disrupting activities, was employed to prepare antimicrobial nanoparticles in conjunction with chitosan (Figure 3e) [160]. The incorporation of rhamnolipid resulted in nanoparticles of smaller and more uniform size, exhibiting a significantly positive surface charge and enhanced stability. The antibacterial efficacy of these chitosan/rhamnolipid NPs against both planktonic and biofilm-state *Staphylococcus* strains was superior to that of either rhamnolipid or chitosan alone, indicating that these hybrid NPs might present a formidable approach for biofilm control.

#### 6.3.3. Dextran Antimicrobial Nanoparticles

Dextran, characterized by α(1→6) glucose-linked polysaccharides with a high degree of linkage variability and branching, is a polymeric material extensively used in drug delivery [161]. Its strong bio-affinity with the polysaccharides in biofilm EPS enhances the penetration of dextran nanoparticles into the biofilm matrix [162]. The abundant active hydroxyl groups and its narrow molecular weight distribution allow dextran to be chemically tailored to suit the needs of nanoparticulate drug delivery more effectively [163]. Barros et al. integrated curcumin into nanoparticles, using PLGA as the hydrophobic core and dextran as the hydrophilic shell [164]. These curcumin-loaded nanoparticles outperformed the free drug in terms of penetration into *Pseudomonas putida* biofilm, resulting in significantly heightened antibiofilm activity. Recognizing dextran’s affinity for the polysaccharides in the biofilm matrix, Li et al. employed cationic dextran to disrupt the intrinsic electrostatic interaction in the biofilm matrix. The biofilm matrix, maintained by the positively charged Pel (positively charged polysaccharide) and eDNA, underwent a gel-to-sol phase transition when disrupted by the cationic dextran, leading to a collapse of the biofilm’s structural integrity (Figure 3f) [165]. This particular form of cationic dextran showed enhanced antibacterial capability against *P. aeruginosa* biofilms, positioning it as a promising cationic substance to combat bacterial biofilm through inducing phase transitions.

#### 6.3.4. Amphiphilic Cationic Copolymer Antimicrobial Nanoparticles

Amphiphilic block copolymers are composed of two or more polymer fragments with distinct solubility properties. These copolymers can spontaneously form various nanostructures in aqueous solutions, such as polymeric micelles, polymersomes, etc. [166] Inspired by the structure of antimicrobial peptides, which consist of hydrophobic and positively charged amino acids, amphiphilic cationic polymers containing hydrophobic segments and positively charged hydrophilic segments were designed as nanomaterials for antimicrobial and antibiofilm treatments. The cationic segments of the polymer are designed to enhance binding to anionic bacterial membranes through electrostatic interactions, while the hydrophobic segments drive the polymer chains to insert into the hydrophobic bacterial membrane, leading to bacterial membrane disruption. Additionally, cationic amphiphilic polymers have been found to inhibit biofilm formation and destroy already-formed biofilm architecture [167]. Takahashi et al. [168] synthesized amphiphilic methacrylate homopolymers PE0 and co-polymers PE31 using reversible addition-fracture chain transfer (RAFT) to control streptococcus mutans biofilm. The minimum biofilm inhibitory concentrations (MBIC) of PE31 and PE0 were 6.3 μg/mL and 8.3 μg/mL, respectively. For established biofilms, the polymer concentration at 1000 μg/mL reduced bacterial biofilm biomass by 80–85%. Zhao et al. synthesized a series of PEG-blocked amphiphilic cationic polymers consisting of hydrophobic alkyl groups and quaternary ammonium salts. They investigated the relationship between the polymer structure and its antibacterial activity against *Escherichia coli* and *Staphylococcus aureus* [169]. The polymers were screened based on PEGylation, the length, and content of hydrophobic alkyl chains, among other factors. The optimized cationic polymer showed excellent antibacterial activity against both *S. aureus* (MIC, 4 μg/mL) and *E. coli* (MIC, 8 μg/mL). Notably, these polymeric nanoparticles can even eradicate bacteria within the biofilm. Vishwakarma et al. [170] synthesized a class of peptidomimetic polyurethanes for bacterial biofilm disruption. These polymers utilized arginine or lysine mimics as cationic groups and phenylalanine and alanine mimics as hydrophobic side groups. These polyurethanes can disrupt biofilms of *P. aeruginosa*, *S. aureus*, and *E. coli*, even those resistant to conventional antibiotics. Moreover, these polyurethanes prevent bacterial attachment to surfaces and enhance bacterial motility, inhibiting biofilm formation in both Gram-positive and Gram-negative bacteria at sub-inhibitory concentrations.

#### 6.3.5. Antimicrobial Peptides Loaded Nanoparticles

Distinct from inorganic NPs and antibiotics, antimicrobial peptides (AMPs), also known as host defense peptides, consist of 12–100 amino acid fragments and exhibit promising antimicrobial efficacy [171]. Found in various organisms, including insects, animals and plants, AMPs serve as a vital defense mechanism in nature. In the lung, AMPs are released by epithelial cells and immune cells into the airway mucus, playing important roles in both innate and acquired immunity [31,172]. Their cationic and amphiphilic nature endows AMPs with broad-spectrum activity against bacteria, fungi, and viruses. The positively charged AMPs can bind to and disrupt negatively charged bacteria cell membranes through electrostatic interaction, leading to intracellular component leakage and subsequent cell death [173]. Therefore, this non-specific bactericidal mechanism positions AMPs as promising antimicrobial agents with a reduced likelihood of resistance development. While over 3000 AMPs have been identified, only seven have receive FDA approval for clinical use [174]. The limited clinical translation might be due to their sensitivity to protease degradation, potential systemic toxicity, and rapid renal clearance post systemic administration. Hence, novel treatment regimens need to be developed to mitigate these limitations.

Inhalation can achieve high drug concentrations locally in the lung, while minimizing systemic drug exposure. This results in decreased toxicity and enhanced efficacy, making it a viable route for AMPs in treating biofilm-associated lung infections. Several nanoparticulate systems, including inorganic and polymeric nanoparticles, have been explored to augment the therapeutic effects and safety profile of AMPs [175,176]. Casciaro et al. used PLGA NPs to encapsulate Esc peptides, AMPs derived from the frog skin, with the objective of enhancing peptide transport through CF mucus and bacterial extracellular matrix [177]. These Esc-peptides-loaded PLGA NPs can be efficiently administered using liquid jet nebulizers available to patients and exhibited enhanced efficacy in inhibiting *P. aeruginosa* growth both in vitro and in vivo. This suggests the potential of PLGA NPs as a reliable delivery system for AMPs targeting the lungs. Additionally, a synthesized branched antimicrobial peptide resistant to biological fluid degradation, has demonstrated efficacy in vitro against numerous Gram-negative multidrug and extensively drug-resistant clinical isolates. Falciani et al. encapsulated SET-M33 in dextran NPs and aerosol-administered these nanoparticles to healthy rat lungs [178]. The findings revealed that these SET-M33-loaded dextran NPs significantly extended the peptide’s lung residence time and effectively managed pulmonary infections in a mouse model afflicted by *P. aeruginosa* induced pneumonia. Collectively, nanoparticulate drug delivery systems show significant promise in delivering AMPs to the lungs to combat both bacteria and the biofilm.

## 7. Nanotechnology-Based Bacterial Biofilm Matrix Degradation Strategy

**Figure 4:**
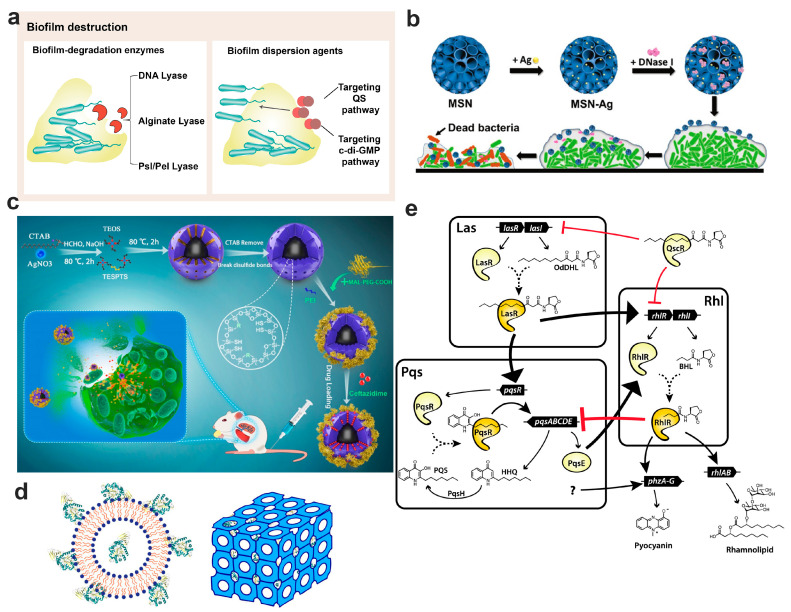
Strategies for biofilm disruption and dispersion to enhance biofilm eradication. (**a**) Schematic overview of the approaches for biofilm disruption and dispersion; (**b**) schematic representation of the preparation of DNase I-loaded MSN-Ag NPs and their role in biofilm eradication (Ref. [183]); (**c**) schematic overview of the design of alginate lyase-fabricated silver nanocomposites for biofilm eradication (Ref. [74]); (**d**) schematic representing the potential differences in PslG loading between the liposomes and LCNP formulations (Ref. [187]); (**e**) overview of quorum sensing circuits in *P. aeruginosa*: large arrows depict primary regulatory pathways between circuits. Solid arrowheads signify positive regulation; flat, red arrowheads denote negative regulation (Ref. [193]).

The EPS matrix of bacterial biofilm acts as a protective barrier. It not only protects the embedded microorganisms from being affected by the harsh environment, but also restrains their access to nutrients. Predominantly comprising proteins, polysaccharides, nucleic acids, and lipids, the EPS facilitates adhesion to substratum and forms a cohesive, three-dimensional polymer network. This structure immobilizes biofilm bacteria and imparts mechanical stability to the biofilm. The composition and characteristics of EPS can differ substantially across biofilms, influenced by factors such as the microorganisms present, experienced shear forces, nutrient availability, and ambient temperature. The dynamic nature of biofilm architecture and its inherent antimicrobial tolerance pose great challenges to conventional antimicrobial interventions, necessitating the development of innovative antibiofilm strategies. A promising approach involves biofilm disruption, leading to direct matrix degradation and the forcible release of embedded bacteria [179]. Once liberated from the protective biofilm matrix, these bacteria become highly vulnerable to antibiotics, facilitating effective biofilm eradication. We will delve into various biofilm disruption strategies (Figure 4a) in the following sections.

### 7.1. Disruption by Biofilm Degradation Enzymes

Disrupting biofilm structure and the polymer network using EPS-specific degradation enzymes can prevent biofilm formation, expose the embedded bacteria to antibiotics, and enhance antibiotic susceptibility. However, the biofilm’s proteases and acidic microenvironment can deactivate these enzymes. Additionally, the EPS-specific enzymes generally lack antibacterial activity and require a combination with antibiotics to eradicate biofilm-associated infections. Nanotechnology has been employed to encapsulate enzymes, shielding them from a hostile environment, ensuring effective delivery to infection sites, and eradicating bacterial biofilm [180].

DNA lyase: Lung inflammation often leads to DNA overproduction within the airway mucus, rendering the mucus highly viscoelastic and challenging to clear. DNA enzymes provide a safe and effective means to ease airway mucus clearance. The inhaled DNA enzyme, Pulmozyme^®^ (Roche, Switzerland), has been clinically used to degrade excess DNA in the airway mucus, thereby reducing mucus viscosity and aiding sputum clearance to improve the pulmonary function of patients with CF [181]. Since DNA is a crucial biofilm component that acts as matrix glue, maintaining the biofilm’s structural integrity, DNA enzymes present a promising solution for degrading and disrupting biofilm structures. Nevertheless, the instability of DNA enzymes limits their application. To address this issue, Tan et al. encapsulated oxacillin and DNase into chitosan NPs to both disrupt biofilm structure and eliminate the embedded bacteria [182]. Compared to other formulations, the oxacillin and DNase co-loaded NPs exhibited superior antibacterial and anti-biofilm activity in vitro, achieving over 98% of biofilm reduction in two days repeated treatment. Yet, the low drug-loading capability of DNase-encapsulated NPs limits their use as antibiofilm arsenals. Dendritic mesoporous silica nanoparticles (MSNs) with large pore sizes offer a solution due to their high drug loading ability and adjustable structure. Combining the biofilm-disrupting power of DNA enzymes with the antimicrobial prowess of Ag NPs, Tasia et al. designed Ag NPs and DNase co-encapsulated MSNs to combat bacterial biofilm infections (Figure 4b) [183]. These MSNs showed potent biofilm dispersion and bactericidal effects against both *Escherichia coli* and *Streptococcus mutans* in vitro.

Alginate lyase: Alginate in the *P. aeruginosa* biofilm acts as a protective shield, defending bacterial biofilm from both immune responses and antibiotic treatments [71]. Alginate lyase can cleave alginate by disrupting the glycosidic bond through a β-elimination reaction, suggesting a potential strategy to enhance antibiotic therapies in managing biofilm infections [184]. Said M et al. employed alginate lyase purified from a marine *Pseudoalteromonas* bacteria alongside antibiotics for bacterial biofilm treatment, noting significant reductions in biofilm biomass produced by a mucoid *P. aeruginosa* strain isolated from patients with CF and improved antibiotic efficacy [185]. Wan et al. designed a core–shell structured nanoparticle comprised of silver nanoparticles and a mesoporous organosilica layer for the co-delivery of alginate lyase and ceftazidime to treat *P. aeruginosa* pulmonary biofilm infections (Figure 4c) [74]. These nanoparticles demonstrated impressive inhibitory and degradation effects in the acidic microenvironment of *P. aeruginosa* biofilm, an ideal condition for drug release and catalytic activity. Moreover, these enzyme and antibiotic co-loaded nanocomposites succeeded in eradicating *P. aeruginosa* from the mouse lungs and minimizing lung injuries, suggesting their potential for clinical use in antibacterial therapies.

Psl/Pel lyase: The extracellular polysaccharides Pel and Psl are crucial elements of the *P. aeruginosa* biofilm matrix, instrumental in the formation and maintenance of biofilm structures and safeguarding embedded bacteria against the host defense and antibiotic threats [69]. Therefore, using the polysaccharide degradation enzymes PelA and PslG to hydrolyze Pel and Psl could weaken the biofilm’s protective matrix, restoring the embedded bacteria’s susceptibility to antibiotics [186]. The half maximal effective concentration (EC_50_) of PelA and PslG for inhibiting biofilm formation were 69.3 and 4.1 nM, respectively, while for disrupting existing biofilm, these values were 35.7 and 12.9 nM, respectively, showcasing their potent biofilm inhibitory and disruptive capabilities. However, the instability of these proteases and the requisite for combined antibiotic therapies curtail their potential as antibiofilm agents, emphasizing the demand for a proficient delivery system. Thorn et al. designed lipidic liquid crystal nanoparticles (LCNPs) co-loaded with PslG and tobramycin to protect PslG from proteolysis, initiate enzyme release at bacterial sites, and amplify overall antimicrobial effects in a *Caenorhabditis elegans* infection model (Figure 4d) [187]. Their results revealed that the LCNPs effectively protect the enzyme from proteolysis, ensured controlled and sustained PslG release, and boosted the antimicrobial effect by 10–100-fold, enhancing the survival rate of *P. aeruginosa* infected *C. elegans*. Consequently, LCNPs emerge as a prospective protective delivery platform for pioneering biofilm-disrupting enzymes combined with antibiotics, augmenting biofilm eradication.

### 7.2. Dispersion the Biofilm through Signaling Pathway

Unlike biofilm disruption, biofilm dispersion is an active event in which sessile, matrix-encased bacteria actively escape from the biofilm, leaving behind an eroded biofilm matrix [179]. Dispersion is the final step of the biofilm lifecycle, which leads to the translocation of the bacteria to new sites for colonization. During dispersion, a single bacterium egresses from the biofilm matrix to resume a planktonic lifestyle. Bacteria in the planktonic state are considered more vulnerable to antimicrobial agents and immune responses. Therefore, triggering biofilm dispersion is viewed as a potential avenue for biofilm control. In this section, we discuss the current knowledge of biofilm dispersion, with a special focus on the two main mechanisms that promote biofilm dispersion.

#### 7.2.1. Biofilm Dispersion Mediated by the Quorum Sensing Pathway

Quorum sensing (QS) is a bacterial cell-to-cell communication process that is contingent on bacterial population density. It is mediated by small diffusible signaling molecules termed autoinducers (AIs). Bacteria release AIs in response to changes in cell density and the species composition of the surrounding microbial community [188]. Many common bacterial pathogens, such as *P. aeruginosa*, *E. coli*, *B. cepacian*, and *S. aureus*, possess QS genes [189]. QS systems are broadly categorized into two groups based on the nature of the secreted Als and their signal transduction modes [190]. Gram-negative bacteria usually use *N*-acyl-homoserine lactones (AHLs) as AIs. These bind to specific intracellular receptor proteins, activating the transcriptional factor and thus regulating the expression of various genes. In contrast, Gram-positive bacteria produce autoinducing peptides (AIPs) that bind to specific transmembrane receptors, leading to targeted gene transcription. As bacterial populations grow, the concentration of AIs increases until reaching a threshold. This initiates the interaction of Als with receptors, inducing the expression of genes pivotal to processes like virulence factor synthesis, motility, and metabolism [55]. In addition, QS significantly influences bacterial resistance by regulating drug efflux pumps and biofilm formation [191]. Therefore, targeting the QS system could be a promising strategy to control biofilm associated infections. Chemicals, such as purine-, phenazine-, triazole-based compounds that disturb the QS system are termed quorum sensing inhibitors. Their mechanisms include inhibiting Als production, degrading Als, and blocking Als binding to receptors [192]. Unlike conventional antibacterial approaches, quorum sensing inhibitors primarily modulate subpopulation behaviors rather than exerting severe selective pressure on the bacteria. This can mitigate the evolution of antibiotic resistance. Quorum sensing inhibitors can be derived from natural sources or synthesized, with many currently under patent-pending status [189].

*P. aeruginosa* uses three intertwined QS circuits: Las, Rhl and Pqs, which regulate the global expression of various virulence-associated genes (Figure 4e) [193]. Notably, the Pqs circuit, which utilizes quinolone metabolites as signaling molecules, is vital in regulating the production of virulence factors, extracellular DNA levels, and biofilm formation. Additionally, the Pqs circuit have been found to make *P. aeruginosa* metabolically less active and significantly reduces the bacteria’s susceptibility to antibiotics [194]. Therefore, Pqs system inverse agonistic compounds hold promise in facilitating biofilm dispersion and diminishing antibiotic resistance [195]. Science quorum sensing inhibitors lack bactericidal activity, they should be used in tandem with antibiotics. For instance, Ho et al. developed self-assembling nanoparticles using synthesized squalenyl hydrogen sulfate. This was to encapsulate both novel lipophilic QS inhibitors and the hydrophilic antibiotic tobramycin [196]. These drug-loaded NPs demonstrated improved mucus layer and biofilm penetration, achieving total biofilm infection eradication with minimal tobramycin doses.

#### 7.2.2. Biofilm Dispersion Mediated by c-di-GMP Pathway

**Figure 5:**
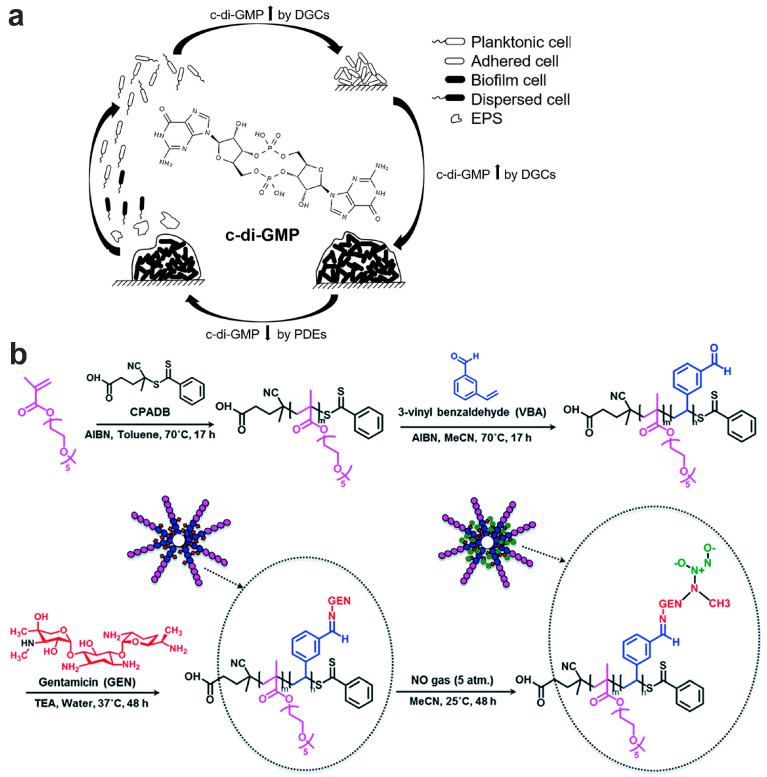
Bacterial biofilm dispersion mediated by the c-di-GMP pathway and the design of nanoparticles containing NO donors to facilitate biofilm dispersion through this pathway. (**a**) Schematic illustration of the role of c-di-GMP signaling molecules in the biofilm lifecycle (adapted with permission from Ref. [179]); (**b**) schematic illustration of the preparation of gentamicin–NONOate NPs via RAFT polymerization (reprinted with permission from Ref. [208]).

As an important intracellular secondary signal molecule, bis-(3′-5′)-cyclic diguanosine monophosphate (c-di-GMP) is crucial in regulating the lifecycle of bacteria, influencing both motility and extracellular matrix production (Figure 5a). In Gram-negative bacteria, c-di-GMP is synthesized by diguanylate cyclases (DGCs) and degraded by phosphodiesterases (PDEs) [179,197]. Elevated c-di-GMP levels encourage a sessile bacterial growth, while reduced levels promote biofilm dispersion, transitioning to a planktonic state and amplifying bacterial motility [198]. Science bacterial lifestyle hinges on c-di-GMP concentrations, modulating DGCs and PDEs’ activity presents a viable approach for biofilm dispersion and antibiotic efficacy enhancement. Low nitric oxide (NO) concentrations have been observed to decrease c-di-GMP levels by interacting with DGCs and PDEs, facilitating biofilm dispersion [51]. Moreover, high NO concentrations exhibited bactericidal effects by disrupting biofilm structures and harming encapsulated bacteria [199]. Gaseous NO, in the form of inhalation, has received approval as an adjunctive therapy. A patient with severe CF witnessed marked improvement in lung function after 46 intermittent inhalations of 160 ppm NO over 28 days [200]. Increasing clinical studies suggest that NO could serve as a potential strategy for biofilm management [201,202]. However, gaseous NO administration requires intricate equipment and meticulous NO concentration monitoring. To circumvent these challenges, low-molecular-weight NO donors like metal nitrosyl complexes, S-nitrosothiols, *N*-diazeniumdiolates, and furoxans were designed [203]. These donors sustainably release NO, inducing biofilm dispersion without inflicting toxicity or physiological impacts. Importantly, responsive-release NO donors can amplify the effectiveness of NO-mediated biofilm dispersion. Ruthenium nitrosyl complexes, for instance, have been identified as viable systems for light-triggered, sustained NO release, resulting in a 50% drop in *S. epidermidis* viability at low NO concentrations [204]. Paired with methicillin, this NO dosage dramatically decreased bacterial resistance, amplifying methicillin sensitivity by a factor of 100. Inspired by the overproduction of beta-lactamase in bacterial biofilm, cephalosporin-conjugated diazeniumdiolates hybrid NO donors were designed to simultaneously release NO and cephalosporin when the β-lactam ring was cleaved by this enzyme [205,206]. Despite the promising performance of these synthetic NO donors in biofilm management, challenges like toxicity and rapid clearance hinder their clinical translation. Therefore, there is an ongoing pursuit for innovative NO delivery systems, optimizing for lower toxicity, controlled NO release, localized NO and antibiotic deployment, and bolstered antibacterial efficacy. Adnan et al. developed an antimicrobial platform in a form of NO gas-releasing polydopamine (PDA)-coated iron oxide NPs (IONPs) [207]. In this design, NO was found to attach to PDA-coated IONPs via a reaction with PDA’s secondary amine groups, forming *N*-diazeniumdiolates (NONOates). An additional polymer, P(OEGMA)-b-P(ABA), boasting hydrophilic and amine pendant groups, was synthesized and grafted onto the PDA-coated IONPs, enhancing these nanoparticles’ colloidal stability. Even at sub-micromolar NO concentrations, these nanoparticles effectively dispersed *P. aeruginosa* biofilms and showcased potent bactericidal activities against both planktonic and biofilm bacteria. Nguyen and colleagues developed NO and gentamicin co-delivery polymeric NPs (GEN-NO NPs) using an amphiphilic block copolymer made of POEGMA and PVBA, further conjugated with a gentamicin–NONOate complex (Figure 5b) [208]. Testing revealed these NPs concurrently released both agents, exhibiting synergistic effects and drastically reducing *P. aeruginosa* viability in both biofilm and planktonic cultures, whereas independent antibiotic and nitric oxide treatments achieved far less reduction. Shen et al. fabricated an amphiphilic polymer (PEO-*b*-PCouNO) that releases NO upon exposure to visible light [209]. This NO-emitting amphiphilic polymer can autonomously form micelles, releasing NO in aqueous medium when exposed to visible light. This photo-induced NO release can effectively disperse *P. aeruginosa* biofilms. These micelles can further co-load with antibiotics (like ciprofloxacin) to simultaneously disperse biofilms and kill bacteria. In summary, low-concentration NO holds potential as an effective tool to enhance biofilm management and the performance of co-delivered antibiotics.

## 8. Nanotechnology-Based Nanoparticle Fabrication Strategies for Bacterial Biofilm Control

**Figure 6:**
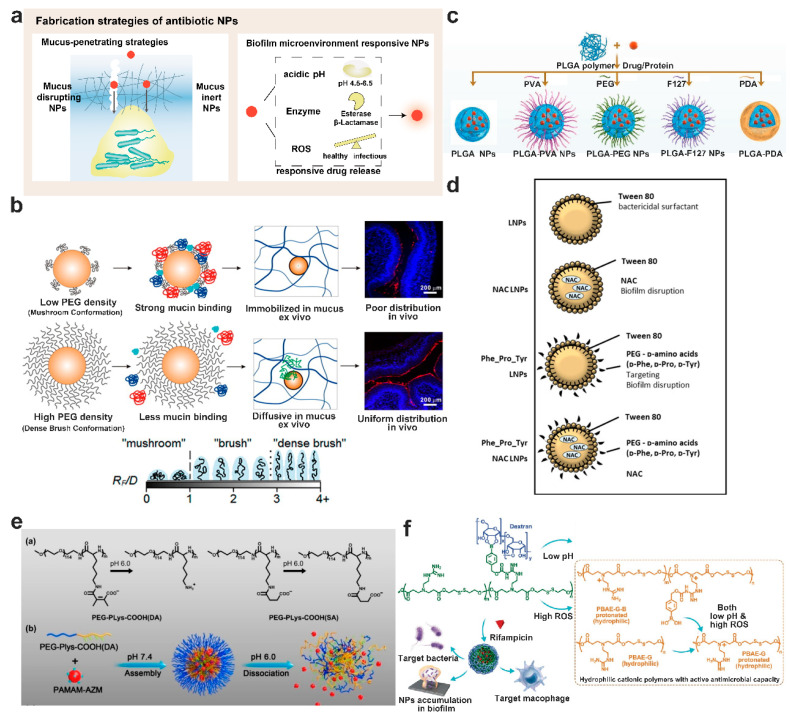
The fabrication strategies of nanoparticles to enhance anti-biofilm efficacy. (**a**) Schematic illustration of strategies to overcome airway mucus and biofilm matrix barriers; (**b**) impact of PEG density on biodegradable NPs transport in mucus and their in vivo distribution (Ref. [30]); (**c**) virous surface modifications of PLGA NPs to investigate their influence on mucus penetrating and cellular uptake (Ref. [218]); (**d**) illustrative overview of functionalized LNPs combined with moxifloxacin as an innovative therapeutic strategy to eradicate bacterial biofilm (Ref. [223]); (**e**) change in chemical structure of DA fabricated PEG-*block*-polylysine (PEG-*b*-Plys) in acidic pH, and the self-assembly of azithromycin-DA NPs at pH 7.4, followed by release of secondary AZM-PAMAM NPs in the acidic bacterial biofilm microenvironment (Ref. [103]); (**f**) schematic depiction of the composition of dextran-coated stimuli-responsive NPs and their antibacterial mechanisms activated by low pH and high ROS at the biofilm sites (Ref. [238]).

Distinct from bacterial biofilms that adhere to substratum, the biofilms in the lung exhibit unique properties. The biofilms of chronic lung infections are typically entrenched in highly viscous mucus or even tenacious sputum, rather than directly on epithelial cell surfaces. Beyond the challenges presented by biofilm barrier, airway mucus also hinders antibiotic-loaded nanoparticles’ access to the biofilm sites. Consequently, airway mucosal barriers should be factored into the design of nanoparticulate drug delivery systems intended for biofilm control. Mucus-penetrating particles serve as an illustrative example of surmounting these mucus barriers, which will be elaborated on in this section (Figure 6a). Additionally, the intricate biofilm structure impedes the penetration of antibiotics or their encapsulated nanomedicines. To tackle this challenge, biofilm-microenvironment-responsive nanoparticles have been developed, offering a promising approach for combating biofilm infections (Figure 6a). These biofilm-microenvironment-responsive NPs enable targeted drug delivery, enhance biofilm penetration, and amplify the antibiofilm efficacy of antibiotic agents while reducing their side effects. In this section, we will further delve into the design strategies for bacterial biofilm-microenvironment-responsive NPs and elucidate their operational paradigm.

### 8.1. Mucus-Penetrating Particles or Muco-Inert Particles

Contrary to common knowledge, local drug delivery through inhalation is an optimal method to address lung infections, as antibiotics or antibiotic-loaded NPs can be directly delivered to the infection sites [210]. However, bacterial biofilm is usually embedded in mucus, which hinders these antibiotics or their nanoparticles from accessing the biofilm. The barrier features of airway mucus result from adhesive and steric interactions within the mucus network [211]. The constituents in the airway mucus can engage with nanoparticles through hydrophobic and electrostatic interactions, inhibiting their deeper penetration into the mucus. Moreover, the entangled mucus network, primarily formed by eDNA and disulfide-bond-linked mucin, creates steric obstructions for particle penetration [52]. In inflammatory and infectious airway diseases, the physicochemical properties of the airway mucus usually differ from those in healthy individuals, complicating the penetration of nanoparticles. Moreover, nanoparticles adhering to the superficial mucus gel layer can be swiftly eliminated by mucociliary clearance [28]. For effective mucus penetration, nanoparticles must resist adhesion by mucus constituents and be adequately small to bypass the dense mucus meshwork. Addressing this, mucus-penetrating particles (MPPs) or muco-inert particles have been designed, showing promise in enhancing drug and gene delivery to mucosal tissues [28,29,212]. Typically, MPPs surfaces are densely coated with hydrophilic polymers, like poly(ethylene glycol) (PEG) or Pluronic 127 (F127), allowing them to minimize adhesion to airway mucus constituents. For instance, dense PEG modification offers a neutral, highly hydrophilic surface which minimizes electrostatic and hydrophobic interactions (Figure 6b). This has been shown to improve mucus penetration [213,214]. Ernst et al. designed tobramycin encapsulated polyester-based particles using PLGA-PEG di-block polymer to overcome mucus and biofilm barriers, enhancing biofilm eradication [215]. The effectiveness of tobramycin against *P. aeruginosa* and *B. cepacia* biofilms was dramatically enhanced when encapsulated under both fluidic and static experimental conditions in artificial mucus. Compared to either free tobramycin or the bulk mixture of tobramycin and blank particles, the MIC of tobramycin-loaded PLGA-PEG NPs against biofilm-embedded *P. aeruginosa* and *B. cepacia* was reduced by more than 1000-fold. Furthermore, PEG-coated particles can be further equipped with moieties to facilitate targeting or cellular uptake. For instance, Tat, a well-researched cell-penetrating peptide, was fabricated onto PEGylated mucus-penetrating nanoparticles for pulmonary delivery of ivacaftor to patients with CF. This dual aim targeted enhancing ivacaftor delivery to airway epithelial cells by rapid diffusion through mucus while simultaneously promoting ivacaftor uptake by the lung epithelial cells [216]. The findings have shown that the presence of Tat on the surface of the MPPs strongly enhanced their uptake by lung epithelial cells. In summary, PEGylated NPs, owing to their excellent biocompatibility, muco-inert nature, and stealth character, are well-documented as a potential strategy to facilitate diffusion through mucosal barriers.

As alternatives, several water-soluble polymers, such as polysarcosine, polyglycydol, poly(vinyl alcohols) (PVA), poly(2-alkyl-2-oxazolines), zwitterionic polymers, and certain hydroxyl-containing polymers, have been explored in their potential in aiding diffusion through mucosal barriers [217]. Characterized by low molecular weight, high hydrophilicity, and non-charged nature, these polymers are promising. Hu et al. developed a PLGA-based platform with various surface modifications, including PEG, PVA, F127, and polydopamine (PDA), and systematically evaluated their mucus penetration ability and cellular uptake (Figure 6c) [218]. Findings revealed that PLGA-PEG and PLGA-F127 NPs showcased superior mucus penetration, while the PDA-modified PLGA NPs excelled in both mucus penetration and cellular uptake. With advancements in controlled polymerization, we can anticipate the emergence of well-defined, low-molecular-weight muco-inert polymers to further the design of cutting-edge mucus-penetrating drug delivery systems.

### 8.2. Enhance Mucus Penetration of Particles by Mucus Disrupting Agents

In addition to the mucus-penetrating particles, mucus-disrupting agents can modify the mucus network, facilitating easier penetration of particles into the mucus. Typically, this strategy employs mucolytic agents, such as using N-acetyl-L-cysteine (NAC), to break the disulfide bond between mucins and to utilize DNA enzymes to degrade extracellular DNA, thereby thinning the mucus. Airway mucus treated with these agents exhibits reduced viscoelasticity and larger mucus mesh pore size, enhancing the penetration of particles into the mucus.

Enzymes can degrade components of airway mucus and have found clinical applications. Among mucus constituents, DNA stands out as a predominant component, entangling with mucin fibers and other mucus ingredients [23]. Elevated DNA levels are observed in inflammatory and infectious lung diseases, primarily due to the necrosis of recruited neutrophils. DNase is routinely used in clinics to treat patients with CF. Recently, DNase-encapsulated nanoparticles have been designed to diminish the crosslinking and viscoelasticity of mucus, thereby aiding particle penetration [219,220,221]. Deacon et al. designed a combination of tobramycin and Dornase alfa (recombinant human deoxyribonuclease Ⅰ, DNase) to concurrently degrade thick DNA-rich mucus and enhance NP penetration into CF sputum [221]. These nanoparticles merge two commonly prescribed CF drugs into a singular nanoparticulate formulation. This represents an innovative approach to surmount the sputum barrier, amplify local drug concentrations, avert systemic side effects, and optimize outcomes for lung infections in patients with CF. In a different study, Suk et al. fabricated a nonviral gene carrier composed of poly-L-lysine conjugated with a 10 kDa PEG segment, either use alone or in combination with mucolytic agents [220]. This synthetic nanoparticulate gene carrier demonstrated superior effectiveness in crossing the mucus/sputum barriers, especially when paired with adjuvant mucolytic treatment using NAC or a combination of NAC and rhDNase.

NAC, a prevalent mucolytic agent, cleaves the disulfide bonds of mucin fibers, leading to decreased mucus viscoelasticity, and promotes drug penetration and that of their nanoparticulate formulations [23]. Dry powder inhalers containing NAC and three distinct antibiotics have been formulated. Research indicates that this combined delivery system of antibiotics and NAC either maintains or boosts antibiotic efficacy, showing significant potential in inhibiting *P. aeruginosa* biofilm formation [222]. Lipid nanoparticles encapsulating NAC were designed with D-amino acids to target and disrupt bacterial biofilms (Figure 6d). These NAC-loaded nanoparticles not only showcased a safe profile than their unloaded counterparts but also exhibited notable biomass and bacterial viability reduction in *P. aeruginosa* biofilms when paired with moxifloxacin. This suggests that such nanoparticulate formulations could serve as potential treatment strategies against *P. aeruginosa* biofilms, either as standalone treatments or in tandem with other antibiotics [223]. Besides NAC, numerous mucolytic agents, including methacholine and thiol-based drugs, have been utilized to enlarge the mucus mesh through disulfide bond disruption, offering potential avenues to bolster nanoparticle mucus penetration [224,225,226]. Thiol-based drugs, in addition to their mucolytic properties, function as antioxidants, either directly via free sulfhydryl groups or by replenishing intracellular glutathione levels [226]. They can also hinder bacterial adherence to respiratory epithelial surfaces and inhibit biofilm formation, thus enhancing antibiotic therapy efficacy. Additionally, a variety of mucolytic enzyme-decorated carrier systems (MECSs) have demonstrated efficiency in cleaving mucus structures, facilitating their journey deeper into the mucus [227]. Studies, both in vitro and in vivo, have highlighted the fact that nanoparticles loaded with these mucolytic enzymes surpass nanocarriers lacking encapsulated enzyme in mucus penetration. Crucially, these mucolytic-enzyme-loaded NPs disrupt mucus structure locally without compromising the overall protective function of the mucosal barrier, hinting at the potential for long-duration treatment using these systems.

### 8.3. Biofilm Microenvironment Responsive Nanoparticulate Systems

Drug-loaded nanoparticles traversing the airway mucus barrier will subsequently encounter the bacterial biofilm and must penetrate the biofilm matrix or release their payloads in a timely manner to achieve a bactericidal effect. Biofilm-microenvironment-responsive NPs have been proven to be a promising strategy for combating biofilm infections [228]. Increasing studies have confirmed that biofilm-microenvironment-responsive NPs enhance biofilm penetration, improve drug targeting efficiency, and enable timely drug release, augmenting the antibiofilm efficacy of the therapeutic agents while minimizing off-target side effects. Characteristics of the biofilm microenvironment, such as acidic pH, overexpression of hydrolases, and hypoxia, can guide the design of these nanoparticles. In addition, nanocarriers leveraging synergistic effects via biofilm-microenvironment-triggered features have proven effective in combating biofilm infections.

Acidic-pH-responsive NPs: Bacterial biofilms typically exhibit an acidic microenvironment (pH 4.5–6.5) due to the accumulation of lactic and acetic acid derived from sugar fermentation. This can guide the design of pH-responsive NPs for site-specific antibiotic release [103,229,230,231]. For instance, Liu and colleagues designed a type of mixed-shell–polymeric micelle (MSPM), comprising a hydrophilic PEG–shell and a pH-responsive poly (β-amino ester). These micelles exhibit a negative charge at physiological pH but become positively charged at pH 5.0 [232]. The stealth properties of the PEG–shell combined with surface charge reversal enable MSPMs to penetrate and accumulate in staphylococcal biofilms. Upon adherence to bacterial surfaces, bacterial lipases degrade nanoparticles, releasing the loaded antibiotics to kill bacteria within the biofilm. Similarly, Yin et al. fabricated ciprofloxacin-conjugated gold nanorods with acidic-induced surface-charge-switchable activity and lipase-triggered drug release properties to combat multidrug-resistant bacterial infections and their biofilms [233]. Gao et al. designed size and charge adaptive azithromycin-conjugated clustered nanoparticles (AZM-DA NPs) for treating bacterial biofilms (Figure 6e) [103]. These particles were formed by electrostatic complexation between azithromycin-conjugated amino-ended PAMAM dendrimer and 2,3-dinethyl maleic anhydride modified PEG-block-polylysine. These particles disintegrate in the acidic biofilm microenvironment, leading to the release of smaller, positively charged AZM-conjugated PAMAM NPs. This release mechanism augments biofilm penetration and bacterial uptake, demonstrating potent anti-biofilm activity.

Biofilm enzymes-responsive NPs: Bacteria excrete various enzymes, such as lipases, phosphatases, phospholipases, and hyaluronidases. These biofilm-specific enzymes offer potential stimuli for designing drug delivering nanocarriers to enhance selective accumulation at microbial infection sites. Although nanoparticles with hydrophilic surfaces enhance their mucus and biofilm matrix penetration, lipophilic surfaces enable bacterial membrane attachment [234]. Wan et al. devised biofilm microenvironment-adaptive NPs with a PLGA core and an enzymatically cleavable TPGS shell to target azithromycin delivery to bacterial biofilms through inhalation [235]. The hydrophobic vitamin E of TPGS enhances biofilm interaction and acts as a prolonged antibiotic release depot, boosting antibiofilm efficacy. As highlighted, lipase can degrade nanoparticles, releasing antibiotics to kill biofilm bacteria after an acidic pH triggers a surface charge switch, collaboratively enhancing biofilm elimination [233].

ROS-responsive NPs: Pro-inflammatory immune cell responses to bacterial biofilm infections results in elevated reactive oxygen species (ROS) levels at infection sites [236]. In *P. aeruginosa* biofilm-associated chronic lung infections, polymorphonuclear leukocytes (PMNs) surround the biofilm, consuming oxygen and producing ROS [16]. The overproduction of ROS at the bacterial biofilm sites serves as a stimulus for designing ROS-triggered nanoparticles. Wang et al. synthesized 4-(hydroxymethyl) phenylboronic acid pinacol ester-modified α-cyclodextrin (Oxi-αCD) as ROS-responsive material to encapsulate moxifloxacin (MXF) for treating pulmonary bacterial infections [237]. With the coating of DSPE-PEG-folic acid, these nanoparticles penetrate sputum easily and target the inflamed tissues. ROS abundance then triggers drug release, displaying enhanced antibacterial efficacy over free drugs and non-targeted counterparts. Ye et al. employed stimuli-responsive nanoparticles loaded with rifampicin to address bacterial resistance (Figure 6f) [238]. These rifampicin-loaded NPs were composed of dextran as the hydrophilic shell and a biodegradable poly (β-amino ester)–guanidine–phenylboronic acid (PBAE-G-B) polymer as the hydrophobic core that can encapsulate the drug. The PBAE-G-B polymer responds to both acidic pH and elevated ROS at the biofilm sites, releasing rifampicin and the cationic polymer. Both agents work synergistically against antimicrobial resistant pathogens. The safety and efficacy of these NPs have been validated in animal models of both Gram-positive and Gram-negative bacterial biofilm infections.

## 9. Biofilm Infectious Models for Evaluation of Antibiofilm Activity

The absence of correlation between the antimicrobial susceptibility of bacterial biofilm cultured in vitro and those formed in vivo has spurred the development of various biofilm models. The reliability of biofilm models is crucial for establishing a correlation between in vitro and in vivo antibiofilm activities, thereby accurately predicting the antibiofilm efficacy of the drug delivery systems. In this section, we will discuss both in vitro and in vivo bacterial biofilm models used to evaluate antimicrobial activity, and their recent advancements in screening antibiotic drug delivery systems.

### 9.1. In Vitro Biofilm Models

**Figure 7:**
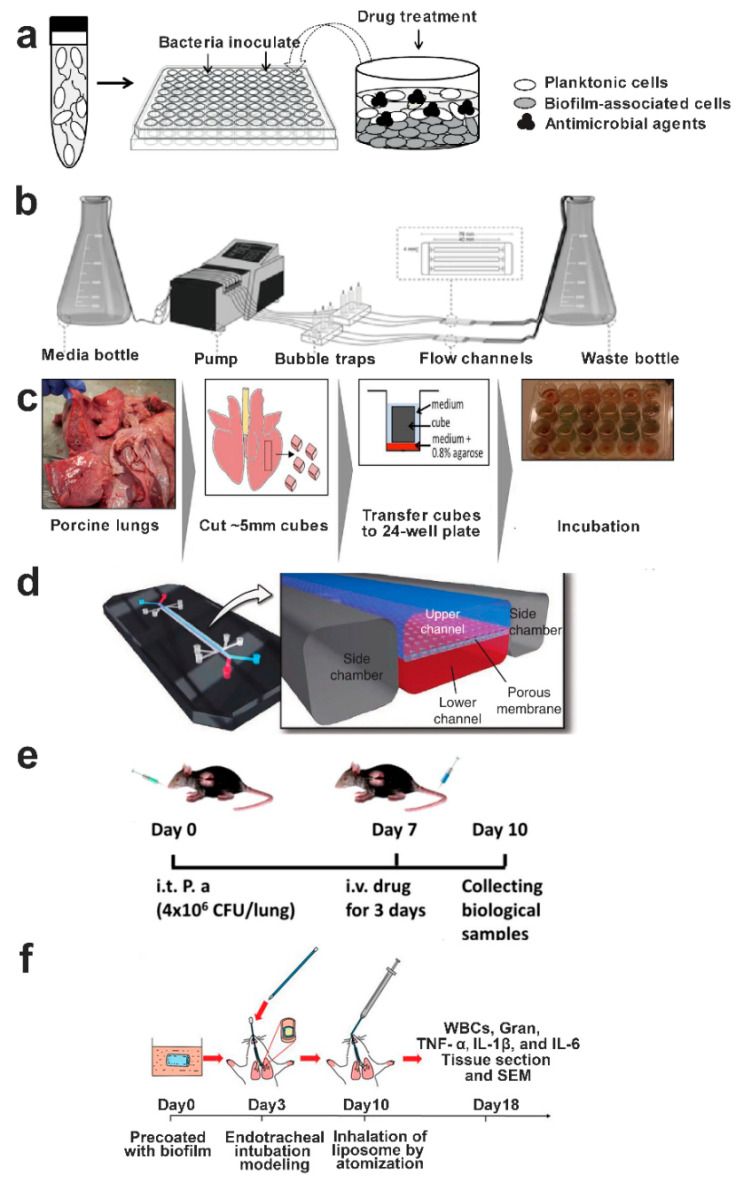
In vitro and in vivo biofilm models for simulating chronic lung infections. (**a**) Schematic representation of static bacterial biofilm construction on plastic (Ref. [240]); (**b**) schematic overview of dynamic bacterial biofilm creation using a flow cell setup (Ref. [242]); (**c**) outline of the protocol for ex vivo infectious lung model using porcine lung (Ref. [244] with modification); (**d**) schematic depiction of the human lung-on-chip microsystem (Ref. [248]); (**e**) timeline detailing the construction of chronic *P. aeruginosa* lung infections in mice and subsequent interventions (Ref. [103]); (**f**) diagram illustrating pharmacodynamic experiments in biofilm-infected rat model (Ref. [35]).

Bacterial biofilm growth models can be classified into static and dynamic systems based on nutrient delivery approaches [239]. Biofilms cultured in static systems offer the benefits of simplicity and are suitable for high-throughput testing. In contrast, dynamic systems provide a closer simulation of the in vivo microenvironment. The microtiter plate method, widely used for testing antimicrobial activity against biofilm, involves culturing bacteria in plastic plates for a set duration. Following this, the culture medium is discarded, and each well is gently washed to remove free-floating bacteria, leaving the bacterial biofilm adhered to the plate (Figure 7a) [240]. For biofilm assessment, crystal violet is commonly employed to stain the biomass, which can then be dissolved to quantify the biomass by measuring the optical density (OD). A significant limitation of this staining method is its inability to differentiate between viable and dead bacteria since the dye stains both equally. Besides, other in vitro biofilm culture methods, such as Calgary biofilm device (CBD) and biofilm ring test (BRT), have been developed to evaluate biofilm’s susceptibility to antibiotics [241].

Dynamic systems, in comparison to static ones, can more faithfully replicate the in vivo conditions by modulating flow, nutrient delivery, and temperature. As depicted in Figure 7b, this system typically consists of a media bottle, peristaltic pump, bubble trap (to negate the impact of bubbles on biofilm growth), flow channel (for biofilm growth), and waste bottle [242]. Biofilms cultured using this approach facilitate pharmacokinetics/pharmacodynamics (PK/PD) studies and allow for real-time microscopic observation. Moreover, this platform can continuously eliminate metabolic waste and planktonic bacteria.

In the context of pulmonary chronic infections, biofilms typically form within airway mucus. This differs from biofilms that form by adhering to substrates, meaning that the previously mentioned standard in vitro biofilm culture methods are unsuitable for respiratory biofilm studies. The incorporation of airway mucus or sputum is essential when constructing this type of mucoid bacterial biofilm. Iglesias et al. investigated the antibiotic activities against *S. aureus* in an in vitro biofilm model, simulating the biofilm structure found in CF sputum [243]. *S. aureus* biofilm was cultured in an artificial sputum medium (ASM), displaying greater elasticity compared to its viscosity and mirroring the characteristics of CF sputum. The team compared antibacterial activity of this setup with biofilm grown in Trypticase soy broth supplemented with glucose and NaCl (TGN). The findings revealed a significant reduction in the potency and efficiency of all tested antibiotics against the ASM-cultured biofilm in comparison to the TGN-cultured one, in terms of viability, metabolic activity, and biomass. This emphasizes ASM’s potential for use in evaluating new therapeutic agents that target biofilms in patients with CF.

In an attempt to simulate in vivo host–pathogen interactions, a three-dimensional A549 lung epithelial cell model was used to culture *P. aeruginosa* biofilm and was compared with the biofilm cultivated on a plastic surface [244]. The data suggested that the *P. aeruginosa* biofilm formed on 3D lung epithelial cells remained resistant to high antibiotic concentrations. This underscores the influence of lung epithelial cells on the antibiotic efficacy against *P. aeruginosa* biofilm, highlighting the importance of host tissues and their surrounding environments for antibiotic performance. Consequently, the 3D lung epithelial model serves as a valuable instrument for testing novel antimicrobial compounds.

Harrison and colleagues developed an ex vivo pig lung model (Figure 7c) which mimics CF lungs to evaluate *P. aeruginosa* growth, quorum sensing effects, virulence factor production, and tissue damage [245]. These ex vivo models, which are cost-effective and ethically acceptable, are also conveniently available and have a storage lifespan of several weeks. As the spatial structure of lung tissue remains intact, both microbial growth and tissue pathologies can be observed through microscopy. Other cutting-edge in vitro biomimetic lung models include organ-on-chip and 3D cell culture methods (Figure 7d) [246,247,248]. Specifically, Si et al. constructed a microfluidic bronchial-airway-on-a-chip technology incorporating pulmonary endothelium and highly differentiated human bronchial-airway epithelium. This platform models viral infection, virulence production, and immune response in humans and holds promise for applications in expediting the assessment of therapeutics and prophylactics with potential antiviral repurposing.

### 9.2. In Vivo Biofilm Models

Although in vitro bacterial biofilm models facilitate rapid, high-throughput analyses of the antimicrobial and antibiofilm efficacies of antibiotics and their nanoparticulate formulations, there is often a gap when translating these findings in vivo. Hence, an in vivo biofilm model is crucial for evaluating the antibiofilm efficiency of antimicrobial agents. Contrary to obstructive pulmonary diseases in humans, animal models do not inherently possess these primary diseases leading to chronic lung infections. As a result, the inoculated bacteria are often rapidly cleared within a few days. Additionally, passive inoculation of animals with a substantial bacterial load makes it challenging to emulate the natural pathological processes of human airways. To more accurately replicate the pathogenesis of chronic lung infections with biofilms, bacteria are embedded in materials like alginate or agarose gel. This shields them from immune system detection, thus extending their infection duration at the inoculation site [103,249]. Gao et al. evaluated the antibiofilm activities of azithromycin-loaded NPs in vivo using a chronic *P. aeruginosa* lung infection model with C57BL/6 mice, recognized as a standard in vivo bacterial biofilm model (Figure 7e) [103]. Specifically, mice were given an intratracheal administration of *P. aeruginosa* (4 × 10^6^ CFU per lung) embedded in alginate microbeads. A chronic lung infection model was effectively established within 7 days. Xie et al. constructed a rat model by immersing central vein catheters into an *S. aureus* suspension for 72 h to initiate biofilm formation (Figure 7f). Tubes pre-coated with the *S. aureus* biofilm were subsequently inserted into the bronchus through the vocal cords, extending to the bronchi [35]. Seven days after intubation, the potential of inhaled berberine hydrochloride-loaded liposomes with varying cholesterol levels to eradicate biofilms was examined. Although experimental animal models reflect the dynamic interactions between bacterial pathogens and host immune response, they fall short in replicating long-term inflammatory responses and extended antimicrobial treatments. The biofilm aggregates in in vivo animal models tend to be small and abundant, in contrast with in vitro biofilms. Therefore, the aforementioned challenges should be acknowledged when devising future animal models, necessitating the creation of increasingly intricate models to accurately represent chronic lung infections.

## 10. Summary and Outlook

Chronic lung infections, resulting from bacterial biofilm, exert a tremendous burden on healthcare systems worldwide. The high mortality and morbidity rates associated with these persistent biofilm-related respiratory infections necessitate the exploration of alternative treatment regimens to offer solutions for these unmet clinical needs. Nanotechnology-based antibiotic drug delivery systems have been proven to be promising in enhancing the efficacy of traditional antibiotics against biofilm infections. These systems achieve this by enabling targeted and localized delivery of high doses of antibacterial agents directly to biofilm sites. This review initially outlined the bacterial biofilm formation processes, the distinct features of the biofilm microenvironment, and the tolerance of biofilm bacteria to antibiotics. Subsequently, we delved into the latest advancements in nanoparticulate drug delivery systems that are tailored for biofilm eradication. This discussion included the typical nanoparticles that are designed for antibiofilm treatment, the agents intended to disrupt or disperse the bacterial biofilm matrix, and the strategies employed to enhance the efficacy of the antibiofilm activity of these nanoparticles. Notably, we introduced biofilm-microenvironment-responsive nanoparticles, where factors like acidic pH, overexpressed enzymes, and ROS are capable of triggering antibiotic release at the biofilm site. This action amplifies antibacterial efficiency while mitigating off-target side effects. Additionally, we underscore the influence of airway mucus or sputum barriers on the delivery efficiency of nanoparticles to bacterial biofilm sites. Overcoming these barriers is crucial for nanoparticles to reach the biofilm and exhibit their antibacterial activities. Finally, we provide an overview of the progress in in vitro and in vivo bacterial biofilm models used to evaluate the therapeutic efficacy of antibacterial agents against pulmonary biofilm infections.

While an increasing body of evidence suggests that nanotechnology offers a promising approach for combating biofilm infections, several challenges remain to be addressed before clinical translation. First and foremost, the potential side effects of these nanoparticles must be closely scrutinized. The therapeutic agents that accumulate at the biofilm sites often constitute a minute fraction of the overall dosage; this is especially the case with intravenous administration [250]. It is widely acknowledged that nanoparticles administered via intravenous injection predominantly accumulate in the liver and spleen, which may lead to pronounced off-target side effects. Therefore, inhalation has emerged as a more direct and dependable administration route for nanomedicines intended to treat bacterial-biofilm-associated respiratory infections. However, the nebulization process might compromise the integrity of nanoparticles, leading to payload leakage. This issue required more thorough investigation. In addition, comprehensive evaluations of nanoparticles’ chronic toxicity are paramount, given the potential interactions with the immune, nervous, and reproductive systems. Secondly, the stability of nanoparticles needs to be thoroughly investigated, including physicochemical stability and in vivo persistence. While many studies focus on the antimicrobial activities of nanoparticulate formulations in direct interaction with bacterial biofilm, the complexities of the bioenvironment mean that in vivo stability must be confirmed before nanoparticles reach their target sites. Thirdly, the intricate designs of many nanoparticles intended for biofilm management often result in challenges with reproducibility. Solutions to facilitate the transition of these nanoparticles from lab to clinical settings are necessary. Microfluidics, capable of enhancing the consistency and control of nanoparticles through precisely manipulating tiny fluid volumes in microchannels, offer a potential solution. The successful launch of mRNA-encapsulated LNPs vaccines against COVID-19 by Pfizer-BioNTech and Moderna demonstrated the feasibility of microfluidics in scaled-up nanomedicines [251]. Lastly, the accuracy of both in vitro and in vivo biofilm models in gauging antibiofilm efficacy is paramount, particularly for biofilm-responsive nanoparticles. Current evidence primarily stems from in vitro biofilm models or in vivo animal models that were initiated by a single bacterium. These models often fail to replicate the intricate bioenvironment found in human beings. Bacterial biofilms in respiratory infections commonly consist of multi-species microcolonies, emphasizing the need to integrate this mixed-biofilm composition when devising models. Innovations such as 3D bioprinting and organ-on-a-chip technologies offer the potential to construct lifelike tissue models in vitro, facilitating disease mechanism investigations and drug screenings [252,253]. These technologies could potentially address the limitations of traditional in vitro and in vivo models in predicting clinical outcomes, bridging the gap between laboratory research and real-world applications.

Instead of directly targeting and eliminating bacteria embedded within biofilms, there is a growing emphasis on the development of anti-virulence agents. These agents, which specifically target quorum-sensing circuits, can substantially mitigate bacterial pathogenicity without impinging on bacterial growth. This approach is quickly gaining traction as a novel therapeutic strategy. Throughout the course of an infection, bacteria routinely secrete a variety of virulence factors that facilitate their adherence and colonization of host tissues. By deploying anti-virulence agents, one can not only attenuate the harmful effects of bacterial biofilms but also circumvent the selective pressures that traditional antimicrobial agents exert on sensitive bacteria. Such a strategy has the added benefit of diminishing the rise of antibiotic-resistant strains. In addition, these agents can bolster the vulnerability of bacteria to the host’s immune defenses and amplify therapeutic outcomes when used in conjunction with conventional antibiotics. Recent research endeavors have identified promising candidates through drug repurposing techniques. Notable examples include niclosamide, clofoctol, and miconazole; all these have demonstrated efficacy in curbing virulence factor production and biofilm establishment across a spectrum of human pathogens [254,255,256]. Collectively, the synergistic application of anti-virulence agents and antibiotics holds significant promise as a treatment modality for biofilm-associated respiratory infections. Furthermore, leveraging nanotechnology as a method of integrating these agents could maximize their collective therapeutic efficacy.

In conclusion, as delineated in the research papers discussed in this review, nanotechnology stands out as a potential tool for utilization in antibiotic delivery systems due to its capacity to effectively challenge bacterial biofilm. Its potential significantly surpasses those of conventional antibiotic therapies. As advancements unfold in material sciences, nanoparticle manufacturing techniques, and the development of more biomimetically inspired biofilm models, we anticipate the emergence of an array of safe, efficacious, and quality-assured antibiofilm nanomedicines. These innovations hold great promise in transforming clinical interventions that can be utilized in treatment of bacterial-biofilm-caused lung infections.

## Figures and Tables

**Table 1:** Pulmonary microorganism-formed biofilms and associated diseases.

Species	Microorganism	Associated Disease	References
Bacteria G^+^	*Staphylococcus aureus*	Ventilator-associated pneumonia (VAP), CF	[35,36]
	*Streptococcus pneumoniae*	Invasive pneumococcal disease	[37]
	*Nocardia*	Pulmonary nocardiosis	[38]
Bacteria G^−^	*Pseudomonas aeruginosa*	CF, COPD	[39,40]
	*Escherichia coli*	VAP	[41]
	*Salmonella*	Typhoid fever	[42]
	*Klebsiella pneumoniae*	COPD	[43]
	*Haemophilus influenza*	COPD	[44]
Fungi	*Aspergillus fumigatus*	Aspergillosis	[45,46]
	*Blastomyces dermatitidis*	Blastomycosis	[47]

## Data Availability

Not applicable.
